# The JNK signaling pathway plays a key role in methuosis (non-apoptotic cell death) induced by MOMIPP in glioblastoma

**DOI:** 10.1186/s12885-019-5288-y

**Published:** 2019-01-16

**Authors:** Zehui Li, Nneka E. Mbah, Jean H. Overmeyer, Jeffrey G. Sarver, Sage George, Christopher J. Trabbic, Paul W. Erhardt, William A. Maltese

**Affiliations:** 1 Department of Cancer Biology, College of Medicine and Life Sciences, University of Toledo, Toledo, Ohio 43614 United States; 2 Center for Drug Design and Development, College of Pharmacy and Pharmaceutical Sciences, University of Toledo, Toledo, OH 43606 USA

**Keywords:** Glioblastoma, Methuosis, Cell death, Chalcones, C-Jun N-terminal kinase, Vacuoles, Endosomes

## Abstract

**Background:**

Synthetic indolyl- pyridinyl- propenones (IPPs) induce methuosis, a form of non-apoptotic cell death, in glioblastoma and other cancer cell lines. Methuosis is characterized by accumulation of cytoplasmic vacuoles derived from macropinosomes and late endosomes, followed by metabolic failure and rupture of the plasma membrane. However, not all IPPs that cause vacuolization are cytotoxic. The main goals of the present study were to identify key signaling pathways that contribute to methuosis induced by cytotoxic IPPs and to evaluate the anti-tumor potential of a prototype IPP in vivo.

**Methods:**

We utilized metabolic flux analysis, glucose uptake, immunoblotting, and selective pharmacological inhibitors to compare the effects of closely related cytotoxic and non-cytotoxic IPPs in cultured glioblastoma cells. To determine whether the use of methuosis-inducing IPPs might be feasible in a therapeutic context, we quantified the distribution of our lead IPP compound, MOMIPP, in mouse plasma and brain, and tested its ability to inhibit tumor growth in an intracerebral glioblastoma xenograft model.

**Results:**

The cytotoxic IPP compound, MOMIPP, causes early disruptions of glucose uptake and glycolytic metabolism. Coincident with these metabolic changes, MOMIPP selectively activates the JNK1/2 stress kinase pathway, resulting in phosphorylation of c-Jun, Bcl-2 and Bcl-xL. At the same concentration, the non-cytotoxic analog, MOPIPP, does not activate these pathways. Pharmacologic inhibition of JNK activity promotes survival, even when cells are extensively vacuolated, but suppression of c-Jun transcriptional activity offers no protection. MOMIPP readily penetrates the blood-brain barrier and is moderately effective in suppressing progression of intracerebral glioblastoma xenografts.

**Conclusions:**

The results suggest that interference with glucose uptake and induction of JNK-mediated phosphorylation of pro-survival members of the Bcl-2 family represent key events in the methuosis death process. In addition to providing new insights into the underlying molecular mechanism of methuosis, the results indicate that compounds of the cytotoxic IPP class may have potential for further development as therapeutic agents for brain tumors.

**Electronic supplementary material:**

The online version of this article (10.1186/s12885-019-5288-y) contains supplementary material, which is available to authorized users.

## Background

Cancer cells often harbor genetic mutations that diminish the expression or function of proteins that regulate apoptosis (e.g., PTEN, p53, Rb) [1, 2]. Consequently, tumors may exhibit reduced sensitivity to therapeutic agents that trigger this classical form of programmed cell death. This limitation has spurred interest in identifying alternative modes of cell death that can be exploited for cancer therapy. Methuosis is one of the most recent additions to the growing array of non-apoptotic cell death mechanisms [3, 4]. Cells undergoing methuosis initially exhibit massive vacuolization of macropinosomes and late endosomes. Our recent studies indicate that this is due to defective trafficking of late endosomes to lysosomes, with concomitant homotypic fusion of the affected vesicular compartments [5]. The defect in lysosome-directed trafficking also affects autophagic flux, with resultant accumulation of autophagosomes [5]. Ultimately, the integrity of the cell membrane is compromised and the vacuolated cells rupture in a manner reminiscent of necrosis. These morphological features are distinct from apoptosis and other non-apoptotic cell death processes, and they cannot be prevented by caspase inhibitors or agents that block necroptosis or autophagy [3].

Methuosis was first described in glioblastoma cells induced to over-express activated forms of the Ras GTPase [6, 7]. Subsequently, our group discovered a series of synthetic chalcones (indolyl-pyridinyl-propenones; IPPs) that can trigger methuosis, independent of constitutive Ras activation [8, 9]. The prototype compound, 3-(5-methoxy-2-methyl-1H-indol-3-yl)-1-(4-pyridinyl)-2-propene-1-one (abbreviated MOMIPP) induces methuosis in glioblastoma (GBM) and other cancer cell lines at low- micromolar concentrations. A recent study provided the first identification of a specific molecular target for MOMIPP; i.e.*,* the phosphatidylinositol-3-phosphate 5-kinase (PIKfyve) [10]. The product of PIKfyve, PI(3,5)P_2_, is known to play a critical role in late endosome trafficking [11, 12]. Since our initial description of methuosis, a number of other reports have noted similar cell death phenotypes promoted by a variety of chemical agents and natural products [13–15]. Features of methuosis have also been described in cells responding to overexpression of miR-199a-3p [16], co-expression of mutant EGFR and K-Ras [17], immunotargeting of CD99 [18], treatment with an oligonucleotide aptamer [19], or NGF-stimulation of TrkA [20].

Despite the growing recognition of the morphological hallmarks of methuosis, the specific molecular mechanisms that link vacuolization of endocytic compartments to loss of cell viability remain poorly understood. Our structure-activity studies of MOMIPP and numerous analogs in GBM cells have provided valuable chemical tools to address this question. Specifically, we found that minor structural modifications of the indole ring yielded a functionally distinct sub-group of IPPs that retained the ability to induce robust morphological vacuolization, with greatly reduced cytotoxicity [21, 22]. By comparing the effects of MOMIPP with one of the non-lethal analogs (MOPIPP; with propyl substituted for methyl at the 2-position of the indole ring), we noted that cells treated with MOMIPP had more severe inhibition of endolysosomal degradation pathways for EGF and LDL receptors [5]. Coincidentally, MOMIPP shows stronger binding affinity (lower K_d_) for PIKfyve than the non-lethal analogs [10], despite the fact that the cells treated with these compounds have similar vacuolated morphologies.

In the present study, the objective was to expand the comparative analysis of cytotoxic versus non-cytotoxic vacuole-inducing IPPs in GBM cells, with the goal of defining pathways essential for triggering cell death. The results indicate that early impairment of glucose uptake and glycolytic metabolism, with attendant activation of JNK signaling and Bcl-2 phosphorylation, are key elements in the methuosis death program.

## Methods

### Cell culture

Human glioblastoma cell lines, U251 (deposited by Darrell Bigner), SF295 (deposited by Paul Kornblith), and SNB19 and SNB75 (deposited by M.L. Rosenblum), were obtained from the Developmental Therapeutics Program (DTP) Tumor Repository, NCI Division of Cancer Treatment and Diagnosis (DCTD) (operated by Charles River Laboratories for the National Cancer Institute, Frederick, MD). The A172 (Cat. No. CRL-1620), LN229 (Cat No. CRL-2611), T98G (Cat No.CRL-1690), and U87MG (Cat No. HTB-14) cell lines were purchased from the American Type Culture Collection (Manassas, VA). Normal human skin fibroblasts were originally derived from a skin biopsy as described previously [23]. All cell lines were maintained in Dulbecco’s modified Eagle medium (DMEM; ThermoFisher, Walthham, MA), supplemented with 10% (*v*/v) fetal bovine serum (FBS) (JR Scientific, Woodland, CA) at 37 °C with 5% CO2/95% air. Cell lines were confirmed negative for *Mycoplasma* contamination by periodic staining with DAPI or use of the PlasmoTest assay (InvivoGen, San Diego, CA).

### Chemicals and antibodies

Indolyl chalcone compounds; MOMIPP [9], MOPIPP [21], 2a and 2q [22] were synthesized as described previously. SP600125 and YM201636 were obtained from Cayman Chemical, Ann Arbor, MI. Bafilomycin-A1, n-methylpyrrolidone (NMP), 5-(N-Ethyl-N-isopropyl)amiloride (EIPA), oligomycin, phloretin, cytochalasin B, carbonyl cyanide 4-(trifluoromethoxy)phenylhydrazone (FCCP) and Solutol-HS15 were from Sigma-Aldrich, St. Louis, MO. XenoLight D-Luciferin-K^+^ salt was obtained from Perkin Elmer, Waltham, MA.

### Immunoblots and antibodies

U251 cells were typically seeded at 1 × 10^6^ cells per 10 cm dish and maintained for 24 h in DMEM containing 10% FBS. On the day after plating, test compounds (or an equivalent volume of DMSO) were added in fresh medium and cells were harvested after the indicated times. Cells were lysed in SDS sample buffer (65 mM Tris-HCl, 10% glycerol, 0.5% β-mercaptoethanol, 2% SDS, pH 6.8) and the protein concentration was determined by colorimetric assay (Bio-Rad, Hercules, CA). Equal amounts of protein (80 μg) were subjected to SDS-PAGE, transferred to PVDF membrane. Immunoblot procedures were described previously [24]. Chemiluminescent signals were quantified using an Alpha Innotech FluorChem HD2 imaging system with Alpha View software.

Antibodies against the following proteins were obtained from Cell Signaling Technology (Danvers, MA): phospho JNK 1/2 (Thr183/Tyr185) (Cat. No. 9251), JNK 1/2 (Cat. No. 9252), phospho-p38 MAPK (Thr180/Tyr182) (Cat. No. 9215), p38 MAPK (Cat. No. 9212S), phospho-Bcl-2 (Ser70) (Cat. No. 2827), Bcl-xL (Cat. No. 2764), phospho-c-Jun (Ser63) (Cat. No. 9261), c-Jun (Cat. No. 9165), phospho-SEK1/MKK4 (Cat. No. 9156), SEK1/MKK4 (Cat. No. 9152), and BAX (Cat. No. 5023). Antibodies against Bcl-2 (Cat. No. sc-7382) and the HA-epitope tag (Cat. No. sc-57,592) were from Santa Cruz Biotechnology, Dallas, TX. The antibody against phospho-Bcl-xL (Ser62) (Cat. No. PA535496) was from ThermoFisher, and the antibody against α-tubulin (Cat. No. T5168) was from Sigma. HRP- coupled goat anti-mouse IgG (Cat. No. 554002) and goat anti-rabbit IgG (Cat. No. 554021) were obtained from BD Biosciences (San Jose, CA).

### Phase contrast microscopy

For live cell imaging studies, 1 × 10^5^ U251 cells were seeded in 35-mm dishes and incubated for 24 h before commencing treatment with compounds. Cells were examined by phase-contrast microscopy using an Olympus IX70 inverted microscope equipped with a heated stage, a DP80 digital camera, and cellSens™ software (Olympus America, Center Valley, PA).

For immunofluorescence localization of Glut1, cells were grown on coverslips, treated with MOMIPP or DMSO for 4 h, washed with PBS, and then fixed in ice-cold methanol. After pre-blocking with 10% goat serum, cells were incubated with anti-Glut1 polyclonal antibody (Millipore, Cat. No. 07–1401) at 1/50 dilution for 1 h, followed by goat-anti-rabbit IgG, conjugated to AlexaFluor-488 (ThermoFisher) at 1/600 dilution. Fluorescence images were captured with an Olympus IX70 inverted fluorescence microscope, using a DP80 digital camera, and cellSens™ software (Olympus America).

### Cell viability

Viability of cells in vitro was assessed by measuring cellular ATP using the CellTiter Glo® luminescence assay according to the manufacturer’s protocol (Promega Corp., Madison, WI). U251 cells were seeded in white-walled opaque 96-well plates (2000 cells/well) with four replicate wells for each culture condition. After addition of compounds at the indicated concentrations, cell viability was assayed at a 48 h endpoint. Luminescence was quantified with a Berthold Tech Centro XS3 LB 960 luminometer, using the preinstalled MikroWin software.

### Metabolic flux analysis

Oxygen consumption rate (OCR) and extracellular acidification rate (ECAR) were determined using a Seahorse XF_P_ analyzer, following standard protocols provided by the manufacturer (Agilent Technologies, Santa Clara, CA). Cell plating densities and concentrations of oligomycin and FCCP were optimized according to the recommended protocols. U251 cells were seeded at 15,000 cells per well in DMEM + 10% FBS in Seahorse miniplates. On the day after seeding, cells in parallel wells were changed over to fresh medium containing either 10 μM MOMIPP or 10 μM MOPIPP, with controls containing equivalent amounts of DMSO. Five hours after addition of the compounds, (or DMSO for controls), the cells were washed and switched to the recommended serum-free base medium for each type of metabolic flux assay. After insertion of the pre-hydrated sensor cartridges, the mini-plates were placed in the Seahorse instrument. OCR was measured under basal conditions (+glucose), and after sequential addition of oligomycin and FCCP. In separate assays, ECAR was measure under basal (glucose-free) conditions, and after sequential addition of glucose and oligomycin.

### 2-deoxy-D-glucose (2-DG) uptake

2-deoxy-D-glucose [1,2-^3^H(N)] (Cat. No. NET549A) was purchased from Perkin Elmer (Waltham, MA). For cells incubated with IPPs for 24 h, cellular uptake of 2-DG was determined essentially as described by Wood et al. [25]. Briefly, U251 cells were plated in 6 cm dishes at 2.5 × 10^5^ cells per dish in standard medium. On the next day, fresh medium was added with the indicated compounds and cells were incubated for 24 h. After removal of medium and washing the cells, the cultures were incubated for 5 min at 37^o^ C in HEPES-buffered saline solution (20 mM HEPES, 140 mM NaCl, 2.5 mM MgSO_4_, 1 mM CaCl_2_, 5 mM KCl, pH 7.4) containing 1 μCi [^3^H]2-DG and 10 μM unlabeled 2-DG. Parallel dishes were incubated under the same conditions, except that 10 μM cytochalasin B was added to block glucose transporter activity. Reactions were stopped by addition of ice-cold 0.9% NaCl, and after three washes, the cells were harvested in 1 ml lysis buffer: 20 mM Tris, 135 mM NaCl, 1 mM MgCl_2_, 1% Triton-X100, pH 8.0. Tritium in the lysates was quantified in a liquid scintillation counter and values for treated vs. control cultures were compared after subtraction of background from the cytochalasin B-treated cultures. To correct for possible differences in cell density after 24 h drug exposure, a portion of each cell lysate was utilized for quantification of α-tubulin by western blot analysis, and the tritium counts were normalized to the tubulin values before being compared to the controls. For short-term drug exposures (2–4 h), differences in cell density were not a factor, so the net tritium uptake values in control and treated cultures were compared directly without normalization to tubulin.

[^3^H]2-DG uptake in freshly isolated mouse erythrocytes was assayed essentially as described by Ohmori et al. [26]. Briefly, erythrocytes were pretreated for 10 min at 37^o^ C in HEPES-buffered saline solution containing 10 μM MOMIPP, 200 μM phloretin + 1 mM HgCl_2_, 100 μM cytochalasin B or DMSO equivalent to the amount added with MOMIPP. Then labeling solution containing 1 μCi [^3^H]2-DG was added and incubation was continued for 5 min. Ice-cold stop solution was added to arrest the reaction and the erythrocytes were pelleted by centrifugation at 4^o^ C. The final pellets were washed twice with stop solution, dissolved in Solvable® (Perkin Elmer), treated with H_2_O_2_ and then subjected to liquid scintillation counting.

### Rubidium uptake

^86^Rb (Cat. No. NEZ072001) was purchased from Perkin Elmer. Ouabain-sensitive ^86^Rb uptake was measured as described by Galuska et al. [27]. U251 cells were plated in parallel wells of a 12-well dish at 50,000 cells/well. On the next day, fresh medium (DMEM + 10% FBS) was added, with 10 μM MOMIPP or an equivalent volume of DMSO. After 4 h the cells were washed and pre-incubated for 10 min in serum-free DMEM, with or without 1 mM ouabain. Finally, 1 μCi of ^86^Rb was added to each culture and incubation was continued at 37 °C for an additional 10 min. Reactions were terminated by washing the cells with ice-cold 100 mM MgCl_2_ and cell lysates were prepared for liquid scintillation counting. The ^86^Rb counts in parallel cultures with ouabain were subtracted from those without ouabain to obtain the net ouabain-sensitive ^86^Rb uptake.

### C-Jun dominant negative construct

The c-Jun dominant-negative (DN) construct, pMIEG3-Jun DN [28], was provided by Alexander Dent (plasmid # 40350; Addgene, Cambridge, MA). The c-JunDN cDNA, which lacks the sequence encoding the first 122 amino acids of c-Jun, was amplified by PCR, using a forward primer: 5′- AAA AAA GGT ACC ATG ACT AGC CAG AAC ACG CTG CCC AGC GTC-3′ to add a KpnI site and a reverse primer: 5‘-AAA AAA-TCT AGA-TCA GCT GGC ATA GTC AGG CAC GTC ATA AGG ATA GCT AAA TGT TTG CAA C-3’ to add an XbaI site. The resulting PCR product was ligated into the pTRE-Tight vector (Clontech, Mountain View, CA), to generate pTRE-Tight (c-JunDN). The latter was combined with pTK-Hyg (Clontech) and introduced into the U251-Tet-On cell line, which stably expresses the reverse tetracycline-controlled transactivator (rtTA) [6], using a Nucleofector™ device and kit T reagents from Lonza (Walkersville, MD). The cells were selected in DMEM containing 10% FBS and 200 μg/ml of both G418 and hygromycin. Single colonies were picked, expanded and tested by western blot analysis for expression of HA-tagged c-JunDN in response to addition of 1 μg/ml doxycycline to the medium.

### RT-PCR assessment of gene expression

Total RNA was extracted and purified from cultured cells using RNeasy Mini kit, following the manufacturer’s protocol (SA Biosciences/Qiagen, Germantown, MD). cDNA was generated by reverse transcription of 100 ng of total RNA with the RT^2^ first strand kit (Qiagen). RNA and cDNA were quantified and checked for purity (OD 260/280) using a Nano-Drop-1000 spectrophotometer (Thermo Fisher). RT-PCR reactions were carried out using primers for SERPINA3 and GFAP from ThermoFisher and GAPDH primers from Qiagen, using an Applied Biosystems StepOne Plus™ system. After 40 cycles, the ΔC_T_ values for SERPINA3 and GFAP versus the GAPDH standard were calculated. ΔΔC_T_ values indicate the difference between the average ΔC_T_ values for the MOMIPP-treated cells and the controls. The expression fold change was determined as 2^-ΔΔCt^.

### Turnover and brain uptake of MOMIPP in vivo

All animal studies were performed in compliance with United States Public Health Service Policy on Humane Care and Use of Laboratory Animals, under protocols approved by University of Toledo Institutional Animal Care and Use Committee (Reference Number 107491).

Swiss Webster mice (8–10 weeks, female) were obtained from Charles River Laboratories and housed in ventilated cages on a 12 h light-dark cycle. MOMIPP was dissolved in NSP (10% NMP, 15% Solutol-HS15 in PBS) and administered to mice via intraperitoneal (IP) injection at a dose of 80 mg/kg. For pharmacokinetic studies, 3 mice were used for each time point. Mice were euthanized by CO_2_ asphyxiation followed by cervical dislocation at the indicated times after drug injection. Blood was collected by cardiac puncture, held on ice in lithium heparin-coated tubes, and centrifuged at 10,000 x g for 20 min. Plasma samples were stored at − 80 °C until analysis. Brains were placed in vials and frozen in liquid N_2_. Prior to quantification of MOMIPP, the frozen brains were weighed and homogenized 1:9 (*w*/*v*) in RIPA buffer (150 mM NaCl, 1% NP40, 0.5% sodium deoxycholate, 1% SDS, 50 mM Tris, pH 7.5).

To determine the concentration of MOMIPP, samples of plasma (200 μl) or brain homogenate (500 μl) were extracted with 1 ml ethyl acetate at 37 °C for 20 min, followed by centrifugation for 2 min at 16,000×g. 800 μl of the resulting extract was vacuum centrifuged at 30 °C for 1 h and the residue was suspended in 100 μl of chromatography elution solution: 30% (*v*/v) acetonitrile and 0.1% (v/v) formic acid in deionized water. Samples (10 μl) were injected onto a Waters Ascentis Express C18 column (75 × 21 mm, 2.7 μm) with matching guard column on a Waters 2795 HT-Alliance LC Separations Module, and isocratic elution was performed at a flow rate of 0.3 ml/min. MOMIPP was detected via multiple reaction monitoring on a Micromass Quattro Micro Mass Spectrometer (MS) in ESI+ mode with capillary voltage 3.0 kV, source temperature 100 °C, desolvation temperature 400 °C, desolvation gas flow 650 l/h, cone gas flow 40 l/hr., and dwell time 0.2 s. MOMIPP was identified as 293.1 > 95.9 at cone voltage 40 V, collision energy 23 V, and a column retention time of 2.1 min. Nitrogen was used as the carrier gas through the mass spectrometer, while argon was used in the collision chamber.

For plasma standards and quality control calculations, solutions of known amounts of MOMIPP were prepared in methanol and the solution was evaporated at 30 °C for 1 h in a vacuum centrifuge. 200 μl of mouse plasma (Pel-Freez Biologicals, Rogers, AZ) was added to each prepared tube to give six total plasma standard concentrations ranging from 10 to 100,000 nM, and three quality control samples from 30 to 30,000 nM. Brain standards were prepared in the same way, except that 500 μl of brain homogenate from untreated mice was added, and the measured concentrations for brain tissue were corrected for the 10-fold dilution of brain tissue in RIPA buffer during homogenization. Levels of MOMIPP in plasma were based on volume and expressed as nM concentration. Brain homogenate levels were measured based on tissue mass prior to homogenization, so that these concentrations represent nmol/kg tissue. For ease of comparison, tissue densities were approximated as 1.0 g/ml, so that brain levels could also be expressed as nM concentration.

### Anti-tumor efficacy study in an orthotopic xenograft model

Athymic CrTac:NCR-Foxn1<nu> mice (female, 7–8 weeks) were purchased from Taconic Biosciences (Rensselaer, NY) and housed under SPF conditions (three per cage) on a 12 h light-dark cycle in ventilated cages with barrier filters. For initiation of xenografts that could be detected by bioluminescence imaging (BLI), we nucleofected U251 cells with pCMV5neo-Luc, which encodes the firefly luciferase gene excised from pGL3 (Promega, Madison, WI). Clonal selection was performed in medium containing G418. The resulting stable cell line (U251-LUC), was maintained in DMEM supplemented with 10% (*v*/v) FBS and 200 μg/ml G418 and was periodically tested for uniform luciferase by immunofluorescence analysis using a luciferase antibody (Sigma, Cat. No. L2164).

The intracerebral xenografts were established based on the method described by Ozawa and James [29]. Mice were anesthetized by IP injection with a mixture of ketamine (100 mg/kg) and xylazine (7.5 mg/kg) and placed on a warming pad. A 1 cm incision was made down the midline of the scalp. A small hole was drilled in the skull using a 25 g needle at a point 1 mm anterior and 2 mm lateral to the bregma. 4 × 10^5^ cells (suspended in 3 μl of DMEM) were injected into the brain using a 10 μl Hamilton syringe equipped with a 27 g needle, with a cuff placed 3 mm from the tip of the needle to control the depth of injection. The hole in the skull was sealed with sterile bone wax and the skin incision was closed with surgical glue. Post-surgical analgesia was provided with buprenorphine (0.1 mg/kg) administered twice a day for the first two days. Tumor growth was monitored by BLI. All mice had focal BLI in the range of 10^7^–10^8^ photons by the fourth day after tumor cell implantation. Mice were then grouped randomly into control and MOMIPP treatment groups (10 mice for the control and 11 mice for the treatment group; one mouse did not survive after surgery). Sample size was determined using Biostat Power and Precision® software, which indicated that 10 mice per group was sufficient to detect a 25% difference in the mean tumor size between control and treated mice (80% confidence), assuming a standard deviation of 20% in each group. MOMIPP (80 mg/kg, in NSP) or vehicle was administered by IP injection every 24 h for 15 consecutive days. Tumor progression was monitored by BLI on the 7th, 11th, and 15th days after commencement of drug treatment. On the 15th day, all mice were euthanized by CO_2_ asphyxiation, followed by cervical dislocation. Blood was collected by cardiac puncture and utilized for blood chemistry analysis with a comprehensive diagnostic profile kit and a VetScan VS2 Analyzer (Abaxis, Union City, CA), following protocols recommended by the manufacturer.

### Statistics

GraphPad Prism software was used for statistical analyses. Student’s unpaired t-test was applied to cell culture studies and Mann-Whitney unpaired test was used for in vivo tumor studies. *P* values ≤ 0.05 were regarded as significant.

## Results

### Differential effects of cytotoxic versus non-cytotoxic IPP’s on glycolytic metabolism

**Fig. 1:**
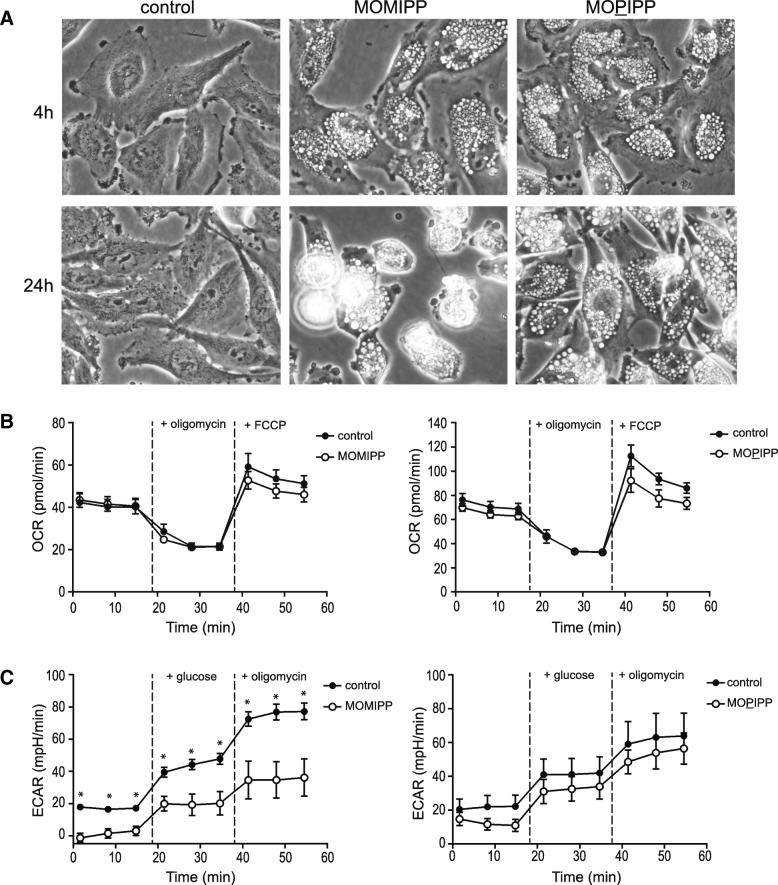
MOMIPP selectively impairs glycolytic metabolism compared to the non-cytotoxic analog, MOPIPP. **a** One day after plating, U251 cells were treated with 10 μM MOMIPP or MOPIPP and examined by phase contrast microscopy after the indicated periods. **b** & **c** Five hours after addition of 10 μM MOMIPP or MOPIPP, U251 cells were subjected to metabolic flux analysis to assess oxidative function (OCR) or glycolytic function (ECAR), using a Seahorse® system, as described in the Methods. Three control and three drug-treated cultures were sampled at each time-point. Values are means (± SD), and significance of the differences between the readings at each point was assessed by Student’s unpaired t-test. In panel (**c**), the asterisks indicate that the ECAR values from the MOMIPP-treated cells were significantly different from the control values at all time points (*p* ≤ 0.05). The ECAR values for the MOPIPP-treated cells were not statistically different from the controls

As shown in Fig. 1a, the morphological effects of the cytotoxic MOMIPP and the non-cytotoxic 2-propyl analog, MOPIPP, are indistinguishable at 4 h, with both compounds inducing extreme vacuolization of U251 GBM cells. However, as noted previously [5], by 24 h the cells treated with MOMIPP begin to round up, detach from the surface of the dish, and lyse, whereas the cells treated with MOPIPP remain attached and viable. Loss of cell viability caused by methuosis-inducing IPPs coincides with a decline in cellular ATP [5, 8]. Therefore, to extend our comparison of the cellular effects of MOMIPP versus MOPIPP, we asked whether MOMIPP might have selective early effects on cellular metabolic functions in GBM cells. To assess metabolic activity, we began by conducting a Seahorse® metabolic flux analysis starting 5 h after addition of the compounds, measuring oxygen consumption rate (OCR) as an indicator of mitochondrial respiratory capacity (Fig. 1b). The results indicated that basal respiration, proton leak (+oligomycin) and maximal respiration (+FCCP) were not substantially different in cells treated for 5 h with MOMIPP or MOPIPP, compared to controls. However, when glycolytic function was assessed by measuring extracellular acidification rate (ECAR), the results indicated that cells treated with MOMIPP for 5 h experienced a significant reduction in basal and glucose-stimulated glycolysis, as well as maximum glycolytic capacity (+oligomycin) (Fig. 1c). In contrast, glycolytic function in the cells treated with MOPIPP was not significantly different from the control (Fig. 1c).

**Fig. 2:**
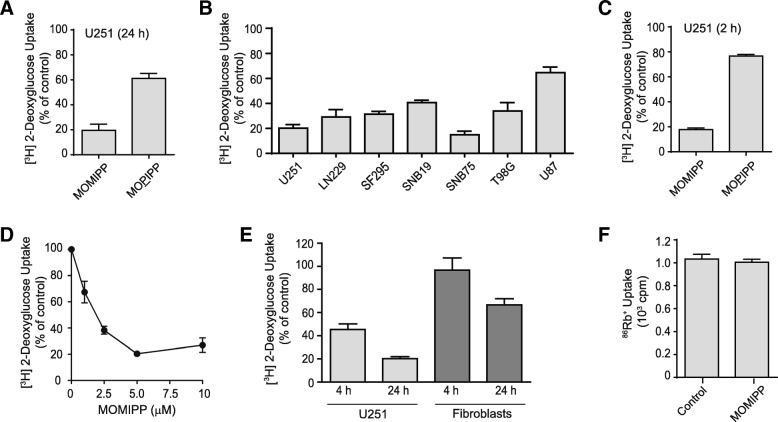
MOMIPP selectively impairs glucose uptake compared to the non-cytotoxic analog, MOPIPP. **a** One day after plating, U251 cells were treated with 10 μM MOMIPP, 10 μM MOPIPP, or DMSO (control). Twenty four hours after addition of the compounds, the cellular uptake of [^3^H]2-DG was assayed as described in the Methods. The results are the mean (± SD) of determinations performed on three cultures, expressed as percent of the mean for the controls. **b** Uptake of [^3^H]2-DG was measured in multiple validated human glioblastoma cell lines, 24 h after addition of 10 μM MOMIPP. The results (mean ± SD, *n* = 3) are expressed as percent of the parallel controls. **c** [^3^H]2-DG uptake was assessed in U251 cells after a 2 h exposure to 10 μM MOMIPP or MOPIPP, with the results expressed as percent of the parallel DMSO controls (mean ± SD, *n* = 3). **d** A dose-response study was performed with U251 cells, measuring [^3^H]2-DG uptake 4 h after addition of the indicated concentrations of MOMIPP, with the results expressed as percent of the controls without drug (mean ± SD, *n* = 3). **e** Uptake of [^3^H]2-DG was compared in U251 cells and normal human skin fibroblasts at 4 h and 24 h after treatment with 10 μM MOMIPP. The results (mean ± SD, n = 3) are expressed as percent of the values in parallel controls treated with vehicle only (DMSO). **f** One day after plating, U251 cells were treated with 10 μM MOMIPP or an equivalent volume of DMSO for 4 h, and ^86^Rb uptake was measured as described in the methods (mean ± SD, *n* = 3)

The glycolytic pathway plays a key role in meeting the bioenergetic needs of glioblastoma and other cancer cells [30]. Therefore, the results of the preceding metabolic flux analysis prompted us to look more closely at glucose transport in cells treated with MOMIPP versus MOPIPP. We found that uptake of the non-metabolizable glucose analog, [^3^H]2-deoxyglucose (2-DG), was reduced by 80% in U251 cells exposed to 10 μM MOMIPP for 24 h (Fig. 2a). By comparison, MOPIPP had a much more modest effect, with an approximate 40% reduction at that time. The effect of MOMIPP on 2-DG uptake was not specific to U251 cells, as variable degrees of suppression were observed in a broad spectrum of human GBM cell lines (Fig. 2b). Since glycolytic function was compromised by MOMIPP within 5 h (Fig. 1c), we repeated the study of 2-DG uptake after only 2 h of drug treatment (Fig. 2c). The results show that suppression of 2-DG is a very early effect of MOMIPP, but not MOPIPP. A dose-response study with MOMIPP indicated that 2-DG uptake was markedly suppressed at ≥2.5 μM (Fig. 2d), which correlates with the GI_50_ previously reported for MOMIPP in U251 cells [22]. Given that MOMIPP suppressed 2-DG uptake in a variety of GBM cell lines, we asked whether 2-DG uptake might be affected to the same extent in non-transformed cells. We previously showed that, compared to glioma cells, cultured human skin fibroblast are less sensitive to the cytotoxic effects of MOMIPP [9]. Therefore, in Fig. 2e we assessed the effects of 10 μM MOMIPP on 2-DG uptake in fibroblasts vs. U251 cells after 4 h or 24 h. The results demonstrate that 2-DG uptake in the fibroblasts was not inhibited at 4 h, and was decreased by only 30% at 24 h (compared to 80% inhibition in the U251 cells). Finally, to rule out the possibility that the effect of MOMIPP on 2-DG uptake was due to generalized disruption of cell membrane structure or function, we assayed the activity of Na^+^,K^+^-ATPase by tracking the ouabain-sensitive uptake of ^86^Rb^+^ (Fig. 2f). The results indicated that 10 μM MOMIPP had no effect on the sodium pump.

**Fig. 3:**
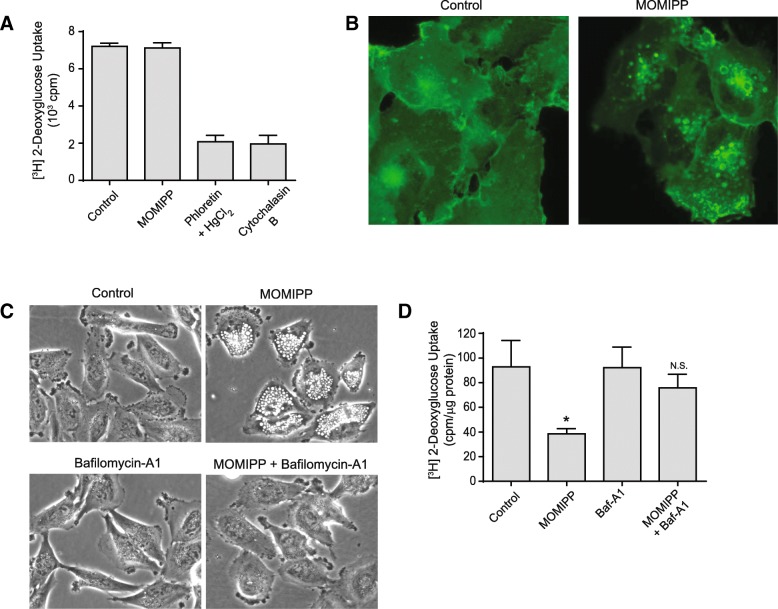
Decreased glucose uptake caused by MOMIPP is not due to direct inhibition of Glut1 and is dependent on endosomal vacuolization. **a** [^3^H]2-DG uptake was assayed in mouse erythrocytes, in the presence of MOMIPP, phloretin + HgCl_2_ or cytochalasin B, as described in the Methods. Assays were performed in triplicate. **b** Immunofluorescence localization of Glut1 was performed in U251 cells 4 h after addition of 10 μM MOMIPP or DMSO (control), as described in the methods. **c** U251 cells were treated with 10 μM MOMIPP, 100 nM Bafilomycin A1, or a combination of both compounds, and cell morphology was examined after 4 h. **d** [^3^H]2-DG uptake was measured in cells treated as in panel C. Each value is the mean (± SD) derived from three separate cultures. *p < 0.05 compared to control. N.S.; not significantly different from Bafilomycin A1 alone

To explore further the mechanism of MOMIPP suppression of 2-DG uptake, we considered the possibility that the compound might directly inhibit glucose transporters or, alternatively, promote sequestration of glucose transporters in the intracellular vacuole compartments. For this purpose, we utilized erythrocyte preparations, which lack endocytic machinery and contain abundant Glut1in their surface membranes. As shown in Fig. 3a, MOMIPP had no direct effect on 2-DG uptake in erythrocytes, while known inhibitors of Glut1 were very effective in reducing 2-DG uptake. On the other hand, immunofluorescence localization of Glut1 in U251 cells revealed prominent sequestration of the transporter in intracellular vacuoles, contrasting with control cells where Glut1 fluorescence was predominantly associated with the cytoplasm and the plasma membrane (Fig. 3b). To confirm that inhibition of 2-DG uptake was related to the formation of endosomal vacuoles, we took advantage of the previously reported ability of the H^+^-ATPase inhibitor, Bafilomycin A_1_, to prevent the formation of endosomal vacuoles initiated by IPPs and other compounds [8, 31]. The results in Fig. 3c&d demonstrate that Bafilomycin A_1_ effectively blocked vacuolization induced by MOMIPP, and prevention of vacuole formation abrogated the inhibitory effect of MOMIPP on 2-DG uptake.

### Cytotoxic IPPs selectively activate the JNK stress kinase pathway

**Fig. 4:**
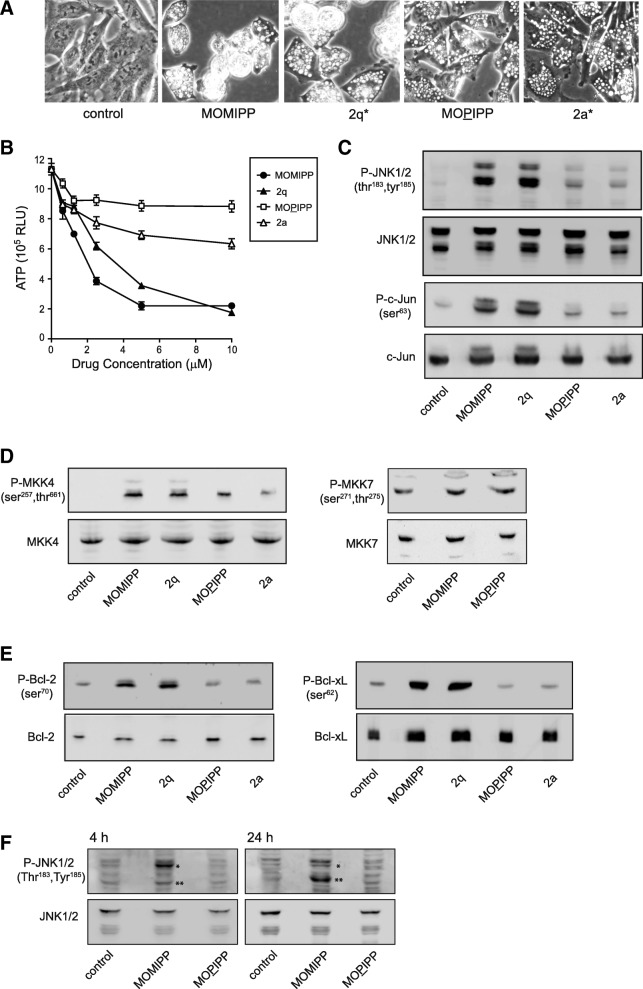
Methuosis-inducing IPPs specifically trigger activation of the JNK pathway. **a** U251 cells were treated with the indicated IPPs at a concentration of 10 μM and phase contrast images were obtained after 24 h. **b** Dose-response studies were carried out with each IPP. Cell TiterGlo® viability assays were performed after incubation for 48 h with compounds at the indicated doses. **c**-**e** U251 cells were treated with the indicated IPPs for 24 h, and immunoblot analyses for the indicated proteins were performed on equal amounts of cellular protein as described in the Methods. **f** U251 cells were treated with the MOMIPP or MOPIPP for either 4 h or 24 h, and equal amounts of cell protein were immunoblotted for phospho-JNK or total JNK as described in the Methods. The 54 kDa (*) and 46 kDa (**) splice variants of phospho-JNK1/2 are indicated by the asterisks. All blots are representative of similar results obtained in three separate experiments

A number of studies have established that cancer cells are particularly susceptible to oxidative stress and cytotoxicity induced by glucose deprivation or inhibition of glycolysis [32]. Activation of the c-Jun N-terminal kinase (JNK) signaling pathway is a key step in this process [32–34]. Thus, we asked whether the cytotoxic methuosis-inducing IPPs might have differential effects on JNK, compared to closely related IPPs that induce vacuolization without cytotoxicity. For this purpose, we selected two methuosis inducers, MOMIPP and 2q, and two non-toxic vacuole-inducers, MOPIPP and 2a. The structures and activities of 2q and 2a were reported previously [22]. As shown in Fig. 4a&b, 10 μM MOMIPP and 2q caused substantial vacuolization, cell rounding and detachment, with loss of viability (ATP) by 48 h. In contrast, cells treated with 10 μM MOPIPP or 2a were vacuolated but remained attached, and they showed relatively small decreases in ATP compared to the vehicle-treated control. When JNK activation (phosphorylation) was assessed by western blot analysis after 24 h (Fig. 4c), we found that MOMIPP and 2q induced major increases in JNK1/2 phosphorylation. By comparison, the JNK phosphorylation signals on the blots from cells treated with the non-toxic MOPIPP and 2a were much weaker. Consistent with the activation of JNK, we also observed a parallel increase in phosphorylation of the JNK target, c-Jun, in cells treated with MOMIPP and 2q, but not MOPIPP and 2a (Fig. 4c).

Two MAP kinase kinases, MKK4 and MKK7, mediate phosphorylation of JNK on Tyr and Thr [35]. These kinases can, in turn, be activated by several different upstream kinases (e.g., ASK1, MLK, TAK1). As shown by the western blots in Fig. 4d, MOMIPP and 2q triggered a robust increase in phosphorylation of MKK4. Treatment with MOPIPP and 2a caused a more modest increase. By comparison, neither MOMIPP nor MOPIPP dramatically altered phosphorylation of MKK7 (Fig. 4d). These observations are consistent with the concept that MKK4 responds predominantly to environmental stress, whereas MKK7 is associated with cytokine-mediated pathways [35, 36].

### Cytotoxic IPPs trigger increased phosphorylation of Bcl-2 and Bcl-xL

In addition to the c-Jun transcription factor, other known substrates for JNK include pro-survival members of the Bcl-2 family, which play key roles in protecting cells from apoptosis and necrosis [37, 38] and regulating autophagy. Indeed, phosphorylation of Bcl-2 and Bcl-xL by JNK1 is postulated to interfere with their interactions with pro-apoptotic members of the Bcl-2 family (e.g., BAX) and the autophagy regulator, Beclin-1, thereby promoting cell death or autophagy [39–41]. The results depicted in Fig. 4e indicate that increased phosphorylation of Bcl-2 and Bcl-xL accompanied the activation of JNK in cells treated with the methuosis-inducing compounds, MOMIPP and 2q.

### Activation of JNK is an early step in methuosis

To determine if activation of JNK represents an initial step in the evolution of the methuosis phenotype, we examined the phosphorylation of JNK after only 4 h exposure of cells to MOMIPP or MOPIPP. The results show an early increase in phosphorylation of JNK upon treatment with MOMIPP, but not MOPIPP (Fig. 4f). Interestingly, the western blots revealed a time-dependent change in the phosphorylation pattern of the JNK splice variants, with the p54 isoform predominating at 4 h (Fig. 4f), and the p46 isoform predominating at 24 h (Fig. 4c&f). Consistent with the activation of JNK, phosphorylation of c-Jun also increased 4 h after addition of MOMIPP (Additional file 1: Figure S1A). In contrast to JNK, there was no detectable change in phosphorylation of the p38 MAP kinase after 4 h of MOMIPP treatment (Additional file 1: Figure S1B). As in the studies conducted at 24 h, we observed substantial increases in phosphorylation of Bcl-2 and Bcl-xL at 4 h in cells treated with MOMIPP (Additional file 1: Figure S1C). However, we did not detect any change in expression or phosphorylation of the pro-apoptotic Bcl-2 family member, BAD (Additional file 1: Figure S1C).

**Fig. 5:**
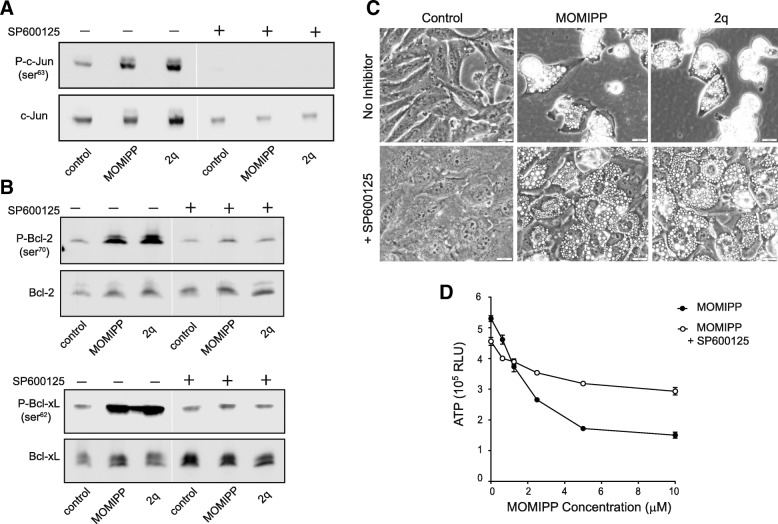
Inhibition of JNK attenuates the cytotoxicity of methuosis-inducing IPPs. U251 cells were treated for 24 h with 10 μM MOMIPP, 2q, or an equivalent volume of DMSO (control), with (+) or without (−) the JNK inhibitor, SP600125 (75 μM). Aliquots of cell lysate containing equal amounts of protein were then subjected to immunoblot analysis for phospho- and total c-Jun (**a**),or phospho- and total Bcl-2 and Bcl-xL (**b**). The blots are representative of similar results obtained in three separate experiments. **c** Cell morphology was assessed by phase-contrast microscopy after a 24-h incubation with MOMIPP or 2q, in the presence or absence of SP600125. **d** U251 cells were incubated for 48 h with the indicated concentrations of MOMIPP, in the presence or absence of 75 μM SP600125, as indicated. CellTiterGlo® viability assays were performed at the 48 h endpoint

An important question raised by the preceding results is whether the activation of JNK is causally related to loss of viability in cells treated with MOMIPP. To address this point we exposed U251 cells to the methuosis-inducing IPPs, MOMIPP and 2q, in the presence or absence of a JNK inhibitor, SP600125 (Fig. 5). The JNK inhibitor completely blocked basal and drug-induced phosphorylation of c-Jun (Fig. 5a). Likewise, the inhibitor prevented the increased phosphorylation of Bcl-2 and Bcl-xL triggered by MOMIPP or 2q (Fig. 5b). Most importantly, addition of SP600125 to cells treated with MOMIPP or 2q had a marked protective effect on cell viability. Although the cells remained extensively vacuolated, the JNK inhibitor prevented them from rounding up, detaching and lysing (Fig. 5c). The protective effect of the JNK inhibitor was also reflected in the results of the CellTiterGlo® viability assay (Fig. 5d).

### Relationship between vacuole formation and JNK activation

The observation that cells treated with MOMIPP or MOPIPP have indistinguishable vacuolated morphologies at early time points, while only the cells exposed to MOMIPP show increased JNK phosphorylation, prompted us to further explore the relationship between JNK activation and endosomal vacuolization.

**Fig. 6:**
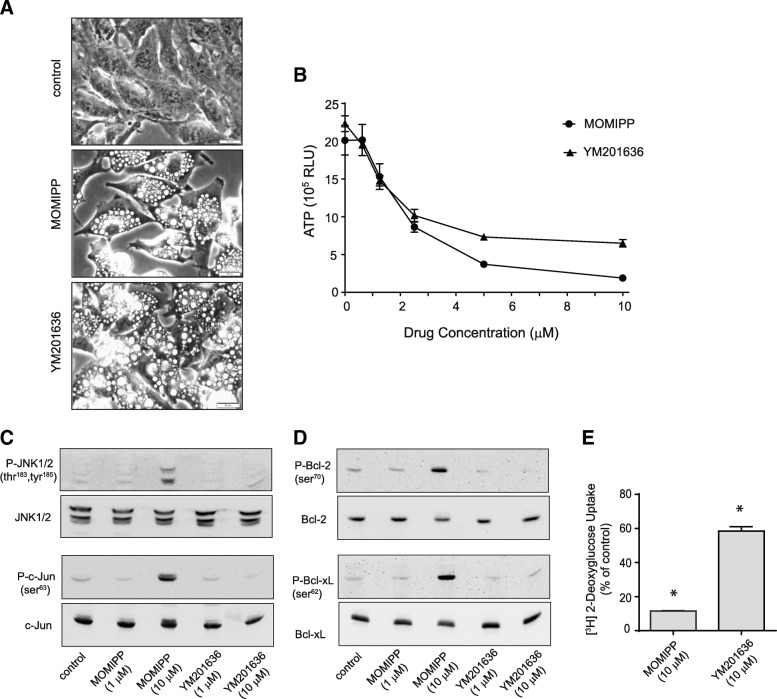
MOMIPP and a PIKfyve inhibitor, YM201636, have different effects on the JNK pathway and glucose uptake. **a** U251 cells were treated for 4 h with 10 μM MOMIPP or YM201636 and cell morphology was observed by phase-contrast microscopy. **b** CellTiterGlo® viability assays were carried out on U251 cells treated with the indicated concentrations of MOMIPP or YM201636 for 48 h (*n* = 3 for each concentration). The decreases in ATP in the cultures treated with both drugs at concentrations of 2.5, 5 and 10 μM (compared to controls without drug) were significant at *p* ≤ 0.05. **c**, **d** U251 cells were treated for 24 h with the indicated concentrations of MOMIPP or YM2011636, and aliquots of cell lysate containing equal amounts of protein were then subjected to immunoblot analysis for phospho- and total JNK1/2 and c-Jun (**c**), or phospho- and total Bcl-2 and Bcl-xL (**d**). The blots are representative of similar results obtained in three separate experiments. **e** [^3^H]2-DG uptake was assayed in U251 cells after a 24 h exposure to 10 μM MOMIPP or YM201636, with the results expressed as percent of the parallel DMSO controls (mean ± SD, *n* = 3). Asterisks indicate that decreases were statistically significant compared to the controls (*p* ≤ 0.05)

We began by asking whether YM201636, a PIKfyve inhibitor with a chemical structure distinct from MOMIPP [42], could also induce JNK activation and subsequent cell death in U251 GBM cells. Within 4 h, 10 μM YM201636 caused extensive cytoplasmic vacuolization, similar to the phenotype induced by MOMIPP (Fig. 6a). By 48 h, YM201636 and MOMIPP both caused obvious decreases in cell viability at concentrations above 2.5 μM (Fig. 6b). However, comparison of the phosphorylation states of JNK, c-Jun, Bcl-2 and Bcl-xL in cells treated with these compounds demonstrated that only MOMIPP promoted substantial JNK activation and phosphorylation of JNK substrates (Fig. 6c&d). Consistent with the potential link between inhibition of glycolytic activity and JNK activation, inhibition of [^3^H]2-DG uptake by YM201636 was only 40% after 24 h, compared to 90% by MOMIPP (Fig. 6e).

**Fig. 7:**
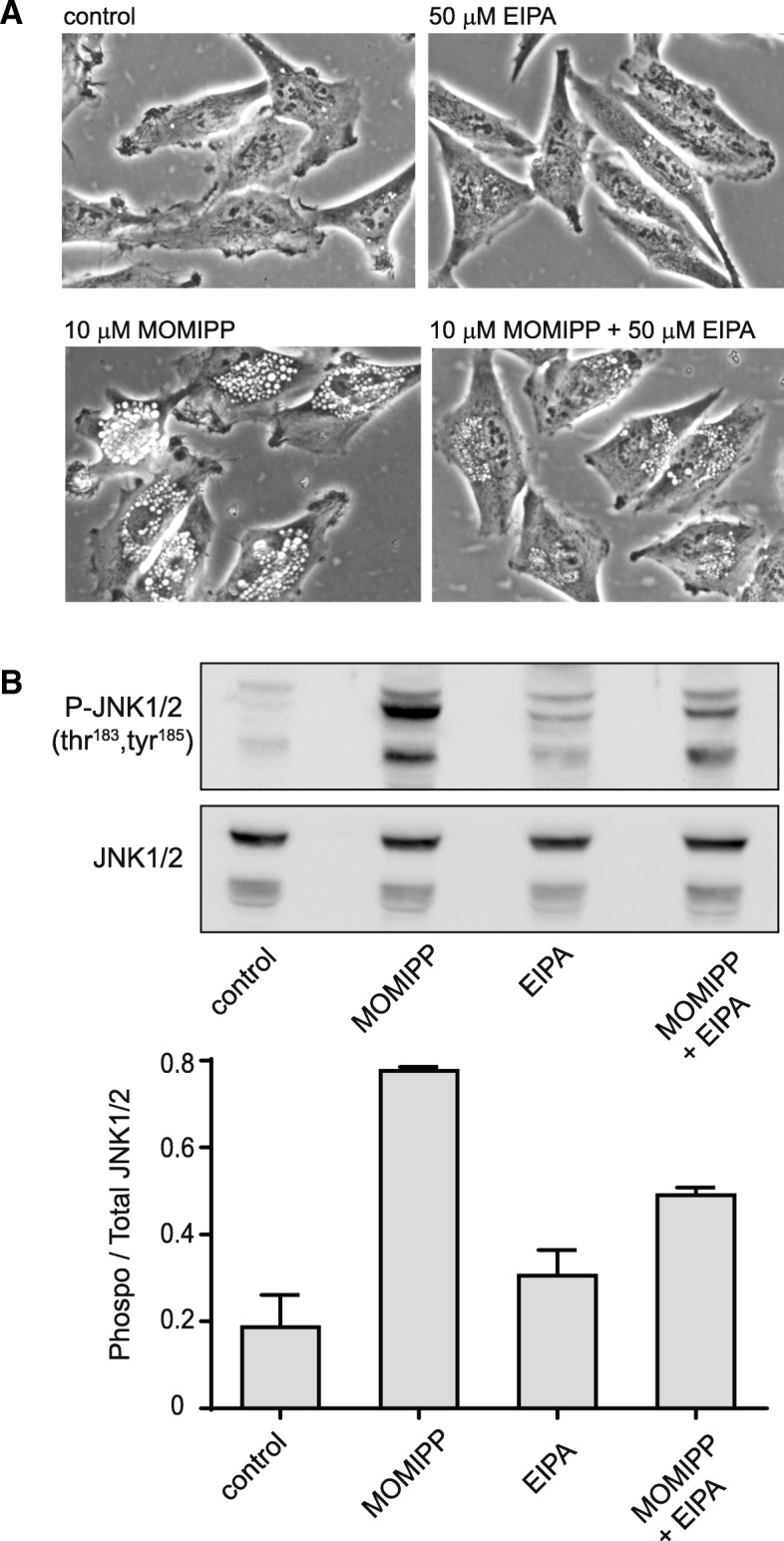
Inhibition of macropinocytosis reduces vacuole formation and attenuates the activation of JNK in cells treated with MOMIPP. U251 cells were treated with MOMIPP, EIPA or a combination of both compounds for 4 h. **a** Cellular vacuolization was assessed by phase-contrast microscopy. **b** Immunoblot analysis was performed as described in the Methods. A representative blot is shown in the upper panel and the ratios of phosphorylated to total JNK (combined 54 kDa and 46 kDa splice variants) from three separate determinations are depicted in the bar graph

In light of the divergent effects of the different vacuole-inducing compounds, we asked whether vacuolization of endocytic compartments was required in order for MOMIPP to induce JNK activation. To address this question, we took advantage of the ability of amiloride and its analogs to inhibit macropinocytosis [43]. As shown in Fig. 7a, treatment of U251 cells for 4 h with 5-(N-ethyl-N-isopropyl) amiloride (EIPA) by itself did not alter baseline cell morphology, but when combined with MOMIPP, EIPA caused a marked decrease in the number and size of vacuoles. At the same time, the 3-fold increase in the ratio of phosphorylated to total JNK triggered by MOMIPP was reduced to just 0.5-fold in the presence of EIPA (Fig. 7b). These results indicate that while not all vacuole-inducing compounds trigger JNK activation, in the case of cells treated with MOMIPP the activation of JNK is indeed linked to the accumulation of vacuoles.

### Activation of c-Jun-mediated transcription is not required for methuosis

**Fig. 8:**
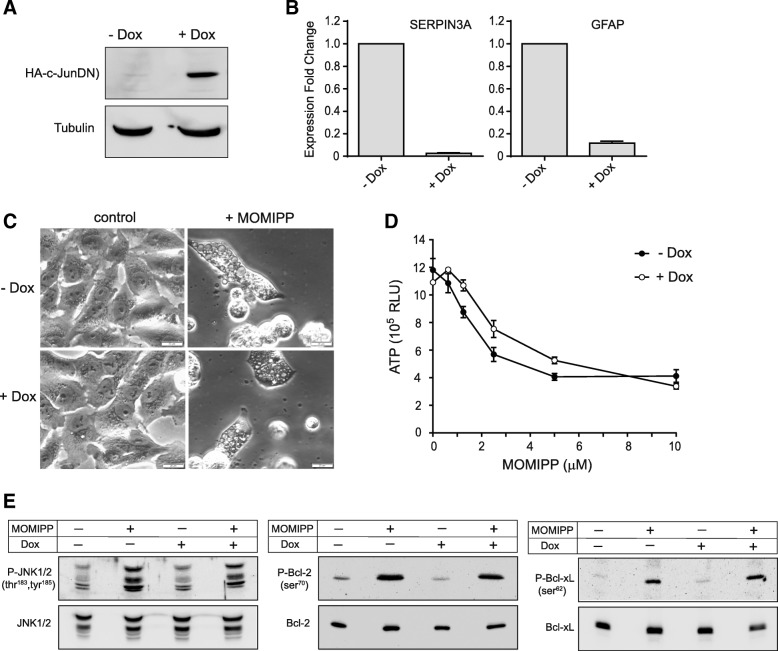
Disruption of c-Jun activity does not prevent MOMIPP-induced methuosis. A stable U251 cell line capable of doxycycline-inducible expression of c-JunDN was developed as described in the Methods. **a** The expression of HA-tagged c-JunDN was verified by immunoblot analysis of cells collected after 48 h in standard culture medium or medium with 1 μg/ml doxycycline (Dox). α-Tubulin served as a loading control. **b** U251-c-JunDN cells were cultured for 24 h in medium with or without Dox, and expression of the indicated genes was quantified by RT-PCR as described in the Methods. The expression fold change is the value: 2^-ΔΔCt^, calculated from three cultures (mean ± SD). **c** U251-c-JunDN cells were cultured for 24 h in the presence or absence of Dox. 10 μM MOMIPP was then added and cell morphology was assessed after 48 h. **d** U251-c-JunDN cells were cultured for 24 h in the presence or absence of Dox and then treated for 48 h with indicated concentrations of MOMIPP (in the presence of absence of Dox). Cell viability was determined with the CellTiterGlo® assay. **e** U251-c-JunDN cells were cultured for 24 h in the presence or absence of Dox. Incubation was then continued for an additional 24 h with or without 10 μM MOMIPP, as indicated. Immunoblot analysis was performed as described in the Methods to detect phosphorylated and total JNK1/2, Bcl-2 and Bcl-xL. Similar results were obtained in three separate experiments

c-Jun functions as part of the AP-1 transcription complex, which controls the expression of many genes involved in cell proliferation, survival and death [44]. In view of the marked increase in JNK-mediated phosphorylation of c-Jun triggered by MOMIPP, we carried out a study to determine if transcriptional events regulated by c-Jun are essential for methuosis. We began by generating a stable U251 cell line (U251-c-JunDN) capable of doxycycline (Dox)-inducible expression of a dominant-negative c-Jun construct. The latter binds effectively to DNA but contains a deletion in the transactivation domain, making it a potent inhibitor of endogenous AP-1 activity [28, 45]. As shown in Fig. 8a, expression of c-JunDN was tightly controlled by Dox in the stable cell line. To confirm that c-JunDN could in fact suppress c-Jun function in U251 cells, we examined the expression of two genes that are regulated by AP-1 transcriptional complexes in astrocytes and glioma cells; SERPINA3 (α_1_-antichymotrypsin) and GFAP [46]. Transcription of both genes was almost completely blocked when c-JunDN expression was induced by Dox (Fig. 8b).

To determine if c-Jun activation plays a key role in methuosis, U251-c-JunDN cells were treated with MOMIPP, with or without prior induction of c-JunDN. The expression of c-JunDN had no effect on the morphological hallmarks of methuosis (e.g., extreme vacuolization, cell rounding, detachment and lysis) (Fig. 8c), and it did not prevent the loss of cell viability, assessed by the ATP assay (Fig. 8d). Western blot analysis confirmed that expression of c-JunDN did not interfere with stress signaling upstream from c-Jun, since JNK phosphorylation was still increased by MOMIPP (Fig. 8e). Consistent with the preservation of JNK activation in cells expressing c-JunDN, the MOMIPP-induced phosphorylation of Bcl-2 and Bcl-xL was also sustained (Fig. 8e).

### Evaluation of MOMIPP pharmacokinetics

The ultimate goal of developing chemical inducers of non-apoptotic cell death in glioblastoma is to employ these compounds as novel therapeutic agents. Therefore, we extended our studies to assess the pharmacokinetic properties of MOMIPP, evaluate its ability to penetrate the blood-brain barrier (BBB), and obtain a preliminary indication of its potential anti-tumor efficacy in a xenograft model.

**Fig. 9:**
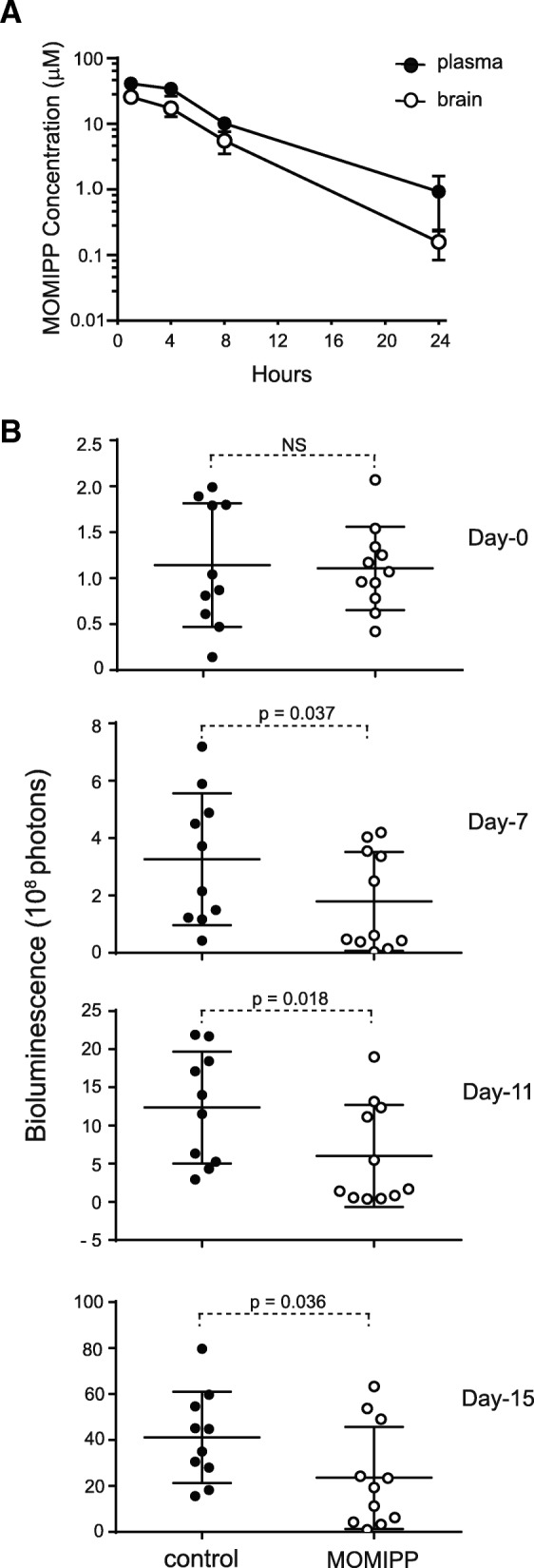
MOMIPP penetrates the blood-brain barrier and inhibits growth of intracerebral U251 xenografts. **a** MOMIPP (80 mg/kg) was administered by IP injection to female Swiss Webster Mice. At each of the indicated time points after injection, blood and brain tissue were obtained from three mice. Plasma and brain tissue were extracted and MOMIPP concentrations (mean ± SD) were determined by LC/MS as described in the Methods. **b** Intracerebral xenografts were established in nude mice by injection of 4 × 10^5^ U251-LUC cells, as described in the Methods. Daily treatment with MOMIPP (80 mg/kg) or NSP vehicle commenced on Day-0 (four days after cell implantation). The study was terminated on Day-15. BLI was performed on Days 0, 7, 11 and 15, and the differences between the control (*n* = 10) and MOMIPP-treated (*n* = 11) groups were evaluated for statistical significance by the Mann-Whitney test for unpaired samples

For initial studies, MOMIPP was prepared in several different solvents and administered via oral (PO) or intraperitoneal (IP) routes at doses ranging from 20 to 80 mg/kg. We learned that the PO route is only effective if mice are fasted, which is impractical for long-term efficacy studies. We also found that MOMIPP is rapidly cleared from the circulation, necessitating high initial doses to achieve sustained plasma levels in the range shown to be effective for inducing methuosis in vitro (≥ 2.5 μM, see Fig. 4b). Based on the results of these preliminary studies, we selected NSP (10% n-methyl-2-pyrrolidone, 15% Solutol HS15, 75% phosphate-buffered saline) as the optimal vehicle and 80 mg/kg as the dose for IP administration of MOMIPP. As shown in Fig. 9a, when mice were given a single injection of the compound, the plasma concentration rapidly reached 40 μM, and remained above 10 μM for 8 h. By 24 h the plasma concentration declined to approximately 1 μM. Of particular interest, the concentrations of MOMIPP in the brain were approximately 50–60% of the concentrations in the plasma up to 8 h, indicating that MOMIPP readily crosses the BBB.

### Effect of MOMIPP on tumor progression

Based on the foregoing studies, we elected to initiate a study to evaluate the effects of MOMIPP on the growth of orthotopic GBM xenografts in immunocompromised mice. The U251 human glioblastoma cell line has been widely used to establish aggressive intracerebral tumors with many of the pathological features of human glioblastoma [47]. To permit monitoring of tumor progression by bioluminescence imaging (BLI), we developed a stable U251 cell line engineered to express firefly luciferase (U251-LUC) (Additional file 2: Figure S2). Four days after intracerebral inoculation of U251-LUC cells, mice were sorted randomly into control and treatment groups (Day-0). At this point BLI indicated no significant difference between the groups (control, 1.14 ± 0.67 × 10^8^ photons; treatment group 1.11 ± 0.45 × 10^8^ photons) (Fig. 9b). Treatment of the mice was then started, with mice in the treatment group receiving daily injections of MOMIPP (80 mg/kg in NSP), while the control group received an equivalent volume of the NSP vehicle. By day-7 the BLI signals in the control group (3.27 ± 0.45 × 10^8^ photons) had tripled compared to day-0, indicating substantial tumor growth. By comparison, tumor progression in the MOMIPP treatment group (1.79 ± 1.72 × 10^8^ photons) was significantly inhibited (Fig. 9b). Subsequent BLI images acquired on day-11 and day-15 (Fig. 9b) confirmed a continued increase in the size of the tumors in the control group, with significant suppression of tumor progression in the MOMIPP-treated group.

The behavior of the MOMIPP-treated mice was not altered during the course of the study. Their weight remained comparable to the controls until day-15, when the controls began to lose weight, prompting termination of the study (Additional file 3: Figure S3). Blood chemistry profiles obtained for all of the control and treated mice at the end of experiment showed no significant differences in key enzymes and metabolites (Additional file 4: Table S1), suggesting that 15-day treatment with high-dose MOMIPP did not cause systemic toxicity or organ failure.

## Discussion

In the present studies, we used MOMIPP and structurally-related indolyl chalcones (IPPs) to explore the mechanism of cytotoxicity underlying the form of non-apoptotic cell death termed ‘methuosis’. The results indicate that although these compounds initially induce morphological phenotypes that appear identical (i.e., massive vacuolization of macropinosomes and endosomes), the cytotoxic compounds differ from the non-lethal compounds in having distinct effects on glycolytic metabolism and the stress-induced JNK signaling pathway. Within the first several hours after vacuoles appear, the prototype methuosis-inducing compound, MOMIPP, caused a substantial reduction in glucose uptake and a decline in glycolytic function. In contrast, the non-cytotoxic analog, MOPIPP did not have major metabolic effects, despite the fact that in previous studies the number of vacuoles per cell was not statistically different between cells treated with MOMIPP or MOPIPP [5]. One possible explanation for these differential effects emerges from the recent identification of the endosomal phosphoinositide kinase, PIKfyve, as a MOMIPP target [10].

The phosphoinositide products of PIKfyve play key roles in the biogenesis of multivesicular endosomes and the assembly of protein complexes required for vesicular trafficking within the endocytic pathway [11, 48]. Indeed, perturbation of the enzyme is known to cause vacuolization of endocytic compartments [49], enlargement and fusion of lysosomes [50], and disruption of autophagic flux [51]. PIKfyve also appears to affect endosome recycling under some circumstances, although this aspect of its function is still not completely understood. For instance, inhibition of PIKfyve disrupts recycling of some junction proteins (claudins) but not others (occludin, E-cadherein) [52]. Of particular relevance for the present study, perturbation of PIKfyve can impair insulin-stimulated glucose uptake and translocation of GLUT4 and GLUT1 from endosomes to the cell surface [53]. It is interesting to note that when Cho et al. [10] compared several IPPs from our series [22], they found that the cytotoxic MOMIPP had a significantly greater affinity for PIKfyve than the non-lethal vacuole inducer, compound, 2a. This raises the possibility that despite similar morphological appearances (i.e., number and size of phase-lucent vacuoles), the cells treated with the more potent PIKfyve inhibitors like MOMIPP may experience a more stringent block in some of the vesicular trafficking functions regulated by this kinase. This concept is supported by our recent study showing that MOMIPP causes a more severe disruption of EGF-receptor and LDL-receptor lysosomal degradation than the non-lethal MOPIPP [5]. Similarly, it is conceivable that compared to MOPIPP and other non-cytotoxic IPPs, MOMIPP may cause greater retention of glucose transporters in non-recycling endosomal vacuoles, ultimately causing a decrease in glucose uptake. While this mechanism merits further investigation, it is not clear whether it alone could account for the differential cytotoxicity of the IPP series. For instance, our finding that the known PIKfyve inhibitor, YM201636, had a much smaller effect on [^3^H]2-DG uptake than MOMIPP (Fig. 6) leaves open the alternative possibility that MOMIPP causes reduced glycolysis and activation of JNK through interactions with targets other than PIKfyve. In this regard, intriguing possibilities include PFKFB3 and PFKFB4 (6-phosphofructo-2-kinase/fructose-2,6-biphosphatases 3 and 4), which play important roles in regulating glycolytic flux, especially in cancer cells [54, 55]. Recently developed antineoplastic inhibitors of PFKFB3 (3PO and PFK15) have chalcone-like structures reminiscent of MOMIPP, with pyridinyl groups as key elements [56, 57]. Therefore, in the future it will be interesting to determine if MOMIPP can directly inhibit PFKFB3.

The results from this study demonstrate that the JNK signaling axis plays a critical role in methuosis, a unique form of cell death induced by MOMIPP and other cytotoxic IPPs. Stress-induced activation of the JNK signaling pathway is widely recognized as a stimulus that can lead to apoptosis [35, 58]. However, previous studies from our group and others have established that methuosis is distinct from apoptosis, based on absence of classical apoptotic hallmarks (e.g., chromatin condensation, DNA laddering, cell shrinkage) and insensitivity to caspase inhibitors [3, 8]. JNK has also has been implicated in regulation of necroptosis, a form of programmed necrosis [58]. But, unlike necroptosis, methuosis is not attenuated by the RIP1 kinase inhibitor, necrostatin-1 (Additional file 5: Figure S4). Thus, methuosis can be added to the growing list of non-apoptotic cell death pathways in which JNK plays a pivotal role [58].

Phosphorylation by JNK increases the activity of c-Jun and other nuclear transcription factors. However, the results of our studies utilizing the c-JunDN construct suggest that MOMIPP-induced cell death does not depend on changes in gene expression mediated by activation of c-Jun. Rather, it appears that post-translational modification of cytoplasmic JNK substrates represents the most likely trigger for cell death. Among the many known JNK targets in the cytoplasm, we focused on Bcl-2 and Bcl-xL, because multi-site phosphorylation of Bcl-2 (T56, S70,T74,S87) and phosphorylation of Bcl-xL (S62) by JNK are thought to disrupt the pro-survival functions of these proteins [39, 59, 60]. The precise mechanism through which Bcl-2 phosphorylation promotes cell death is still unclear, and it may vary depending on the cell type and the stimulus [61]. Possibilities include: 1) disruption of interactions with pro-apoptotic proteins like Bax, facilitating outer mitochondrial membrane pore formation and release of cytochrome c [62]; 2) alteration of Bcl-2 function in the endoplasmic reticulum, causing discharge of Ca^2+^, with consequential mitochondrial calcium overload [63]; 3) release of Beclin-1 from inhibitory Beclin-1/Bcl-2 complexes, causing increased autophagy [41]; or 4) loss of mitochondrial membrane potential and release of proteins like AIF, which are capable of inducing caspase-independent cell death [64, 65]. The latter mechanism merits serious consideration, since methuosis is not blocked by caspase inhibitors [8, 15]. Finally, it should be noted that activation of JNK by MOMIPP could have pleiotropic effects beyond phosphorylation of Bcl-2/Bcl-xL. For example, JNK activation is known to amplify production of reactive oxygen species [66], which has the potential to compromise metabolic functions and trigger necrotic cell death [67].

The molecular events linking endosomal vacuolization and changes in glycolytic metabolism to the activation of JNK remain to be defined. Two upstream kinases, MKK4 (SEK1) and MKK7, are known to phosphorylate JNK, with MKK7 mediating signals from proinflammatory cytokines (e.g., TNF) and MKK4 serving as a sensor for environmental stress [35, 36]. Previous studies have suggested that glucose deprivation may modulate MKK4 by altering the crosstalk between two JNK-interacting scaffold proteins, JIP1 and JIP3, resulting in activation of ASK1, a kinase that targets MKK4 [68]. Therefore, it is conceivable that the JIP1/3 → ASK1 → MKK4 signaling module may serve as a conduit to activate JNK when endosomal trafficking is disrupted and glucose levels fall during the early stages of methuosis. Our finding that MKK4 was robustly activated by the methuosis-inducing IPPs, MOMIPP and 2q, and less so by the non-cytotoxic vacuole-inducers, MOPIPP and 2a, supports this concept.

Thus far, evaluation of the anticancer activity of methuosis-inducing compounds has been confined mainly to cell culture systems. However, two recent studies have highlighted the potential for exploiting this novel form of cell death for treating cancers in vivo. In one study Huang et al. [15] identified a unique 4′6’-disubstituted aza-indole that selectively induced methuosis in a broad panel of cancer cell lines in vitro and suppressed the growth of subcutaneous MDA-MB-231 breast cancer xenografts in immunocompromised mice. In a separate study, Ahlstedt et al. [69] found that a quinolone-based methuosis inducer, Vacquinol-1, reduced the size of brain tumors in syngeneic rat models, although no survival advantage was noted. In the present study, we found that MOMIPP readily penetrates the BBB and significantly suppresses the progression of intracerebral GBM xenografts without overt toxicity in nude mice. However, overall growth suppression was modest, and high doses of MOMIPP were required to compensate for the rapid clearance of the compound from the circulation. Even with relatively high daily doses, our pharmacokinetic observations suggest that by the end of each 24 h period the brain levels of MOMIPP would likely fall below the concentrations found to be therapeutically effective when maintained in vitro. For these reasons, we did not carry out survival studies or immunohistochemical analyses of JNK activity in tumor tissues at this stage. The initial findings reported here, coupled with the fact that MOMIPP can kill GBM cells that are resistant to the standard drug, temozolomide [9], suggest that further development of IPPs as possible therapeutic agents for brain tumors is warranted. Improvements in efficacy may be realized by structural modifications that increase potency or prolong drug half-life in vivo. In addition, the incorporation of these compounds into sustained-release formulations or targeted delivery vehicles may prove advantageous. Finally, the new mechanistic insights pointing to decreased glycolytic function and induction of the JNK stress pathway as important precipitating events in methuosis may suggest opportunities for synergistic combinations with other therapeutic agents that alter these pathways [57].

## Conclusion

These studies provide new insights into the molecular mechanisms underlying methuosis, a non-apoptotic form of cell death that can be induced in glioblastoma and other types of cancer by small molecules. Herein we found that massive vacuolization of endosomal compartments induced by MOMIPP causes an early suppression of glucose uptake and glycolytic metabolism, accompanied by induction of the JNK stress-signaling pathway. These events appear to be pivotal for cell death, since they are not triggered by closely related non-cytotoxic IPPs and pharmacological inhibition of JNK offers substantial protection. Among the consequences of JNK activation, posttranslational events, such as phosphorylation of pro-survival members of the Bcl-2 family, are probably more important than transcriptional events mediated by c-Jun, since a dominant-negative form of c-Jun had no effect on methuosis. Finally, the present studies provide preliminary support for development of IPPs as potential therapeutic agents for brain tumors by showing that the prototype compound, MOMIPP, can readily penetrate the BBB and can inhibit the growth of orthotopic glioblastoma xenografts in mice.

## Additional files

Additional file 1: **Figure S1.** Increased phosphorylation of c-Jun, Bcl-2 and Bcl-xL are early events during MOMIPP-induced methuosis. (DOCX 117 kb)

Additional file 2: **Figure S2.**. Luciferase expression in stable U251-LUC cells. (DOCX 395 kb)

Additional file 3: **Figure S3.** Long-term treatment with MOMIPP does not cause weight loss in nude mice. (DOCX 159 kb)

Additional file 4: **Table S1.** Blood chemistry profiles obtained after treatment of mice for 15 d with MOMIPP or vehicle. (DOCX 13 kb)

Additional file 5: **Figure S4.** MOMIPP-induced cell death is not prevented by a necroptosis inhibitor. (DOCX 471 kb)

## Abbreviations

BBB: Blood-brain barrier
DAPI: 4′,6-diamidino-2-phenyl-indole
DMEM: Dulbecco’s modified Eagle medium, high glucose formulation
DMSO: Dimethylsulfoxide
FBS: Fetal bovine serum
HRP: Horseradish peroxidase
IPP: Indolyl- pyridinyl- propenone
JNK: c-Jun N-terminal kinase
MOMIPP: 3-(5-methoxy-2-methyl-1H-indol-3-yl)-1-(4-pyridinyl)-2-propene-1-one
NMP: N-methyl-2-pyrrolidone
PAGE: Polyacrylamide gel electrophoresis
PBS: Phosphate-buffered normal saline
PVDF: Polyvinylidene difluoride
SDS: Sodium dodecyl sulfate

## References

[1] Ishii N, Maier D, Merlo A, Tada M, Sawamura Y, Diserens A-C, Van Meir EG (1999). Frequent co-alterations of TP53, p16/CDKN2A, p14arf, PTEN tumor suppressor genes in human glioma cell lines. Brain Pathol.

[2] Delbridge AR, Valente LJ, Strasser A (2012). The role of the apoptotic machinery in tumor suppression. Cold Spring Harb Perspect Biol.

[3] Maltese WA, Overmeyer JH (2014). Methuosis: nonapoptotic cell death associated with vacuolization of macropinosome and endosome compartments. Am J Pathol.

[4] Maltese WA, Overmeyer JH (2015). Non-apoptotic cell death associated with perturbations of macropinocytosis. Front Physiol.

[5] Mbah NE, Overmeyer JH, Maltese WA (2017). Disruption of endolysosomal trafficking pathways in glioma cells by methuosis-inducing indole-based chalcones. Cell Biol Toxicol.

[6] Overmeyer JH, Kaul A, Johnson EE, Maltese WA (2008). Active ras triggers death in glioblastoma cells through hyperstimulation of macropinocytosis. Mol Cancer Res.

[7] Bhanot H, Young AM, Overmeyer JH, Maltese WA (2010). Induction of non-apoptotic cell death by activated Ras requires inverse regulation of Rac1 and Arf6. Mol Cancer Res.

[8] Overmeyer JH, Young AM, Bhanot H, Maltese WA (2011). A chalcone-related small molecule that induces methuosis, a novel form of non-apoptotic cell death, in glioblastoma cells. Mol Cancer.

[9] Robinson MW, Overmeyer JH, Young AM, Erhardt PW, Maltese WA (2012). Synthesis and evaluation of indole-based chalcones as inducers of methuosis, a novel type of nonapoptotic cell death. J Med Chem.

[10] Cho H, Geno E, Patoor M, Reid A, McDonald R, Hild M, Jenkins JL (2018). Indolyl-pyridinyl-propenone-induced methuosis through the inhibition of PIKFYVE. ACS Omega.

[11] Shisheva A (2008). PIKfyve: partners, significance, debates and paradoxes. Cell Biol Int.

[12] de Lartigue J, Polson H, Feldman M, Shokat K, Tooze SA, Urbe S, Clague MJ (2009). PIKfyve regulation of endosome-linked pathways. Traffic.

[13] Nara A, Aki T, Funakoshi T, Unuma K, Uemura K (2012). Hyperstimulation of macropinocytosis leads to lysosomal dysfunction during exposure to methamphetamine in SH-SY5Y cells. Brain Res.

[14] Cingolani F, Simbari F, Abad JL, Casasampere M, Fabrias G, Futerman AH, Casas J (2017). Jaspine B induces nonapoptotic cell death in gastric cancer cells independently of its inhibition of ceramide synthase. J Lipid Res.

[15] Huang W, Sun X, Li Y, He Z, Li L, Deng Z, Huang X, Han S, Zhang T, Zhong J (2018). Discovery and identification of small molecules as methuosis inducers with in vivo antitumor activities. J Med Chem.

[16] Minna E, Romeo P, De CL, Dugo M, Cassinelli G, Pilotti S, Degl'Innocenti D, Lanzi C, Casalini P, Pierotti MA (2014). miR-199a-3p displays tumor suppressor functions in papillary thyroid carcinoma. Oncotarget.

[17] Unni AM, Lockwood WW, Zejnullahu K, Lee-Lin SQ, Varmus H (2015). Evidence that synthetic lethality underlies the mutual exclusivity of oncogenic KRAS and EGFR mutations in lung adenocarcinoma. eLife.

[18] Manara MC, Terracciano M, Mancarella C, Sciandra M, Guerzoni C, Pasello M, Grilli A, Zini N, Picci P, Colombo MP (2016). CD99 triggering induces methuosis of Ewing sarcoma cells through IGF-1R/RAS/Rac1 signaling. Oncotarget.

[19] Reyes-Reyes EM, Salipur FR, Shams M, Forsthoefel MK, Bates PJ (2015). Mechanistic studies of anticancer aptamer AS1411 reveal a novel role for nucleolin in regulating Rac1 activation. Mol Oncol.

[20] Li C, Macdonald JI, Hryciw T, Meakin SO (2010). Nerve growth factor activation of the TrkA receptor induces cell death, by macropinocytosis, in medulloblastoma Daoy cells. J Neurochem.

[21] Trabbic CJ, Dietsch HM, Alexander EM, Nagy PI, Robinson MW, Overmeyer JH, Maltese WA, Erhardt PW (2014). Differential induction of cytoplasmic vacuolization and methuosis by novel 2-indolyl-substituted pyridinylpropenones. ACS Med Chem Lett.

[22] Trabbic CJ, Overmeyer JH, Alexander EM, Crissman EJ, Kvale HM, Smith MA, Erhardt PW, Maltese WA (2015). Synthesis and biological evaluation of indolyl-pyridinyl-propenones having either methuosis or microtubule disruption activity. J Med Chem.

[23] Maltese WA, DeVivo DC (1984). Cholesterol and phospholipids in cultured skin fibroblasts from patients with dystonia. Ann Neurol.

[24] Maltese WA, Wilson S, Tan Y, Suomensaari S, Sinha S, Barbour R, McConlogue L (2001). Retention of the Alzheimer's amyloid precursor fragment C99 in the endoplasmic reticulum prevents formation of amyloid beta-peptide. J Biol Chem.

[25] Wood TE, Dalili S, Simpson CD, Hurren R, Mao X, Saiz FS, Gronda M, Eberhard Y, Minden MD, Bilan PJ (2008). A novel inhibitor of glucose uptake sensitizes cells to FAS-induced cell death. Mol Cancer Ther.

[26] Ohmori T, Adachi K, Fukuda Y, Tamahara S, Matsuki N, Ono K (2004). Glucose uptake activity in murine red blood cells infected with Babesia microti and Babesia rodhaini. J Vet Med Sci.

[27] Galuska D, Pirkmajer S, Barres R, Ekberg K, Wahren J, Chibalin AV (2011). C-peptide increases Na,K-ATPase expression via PKC- and MAP kinase-dependent activation of transcription factor ZEB in human renal tubular cells. PLoS One.

[28] Wang ZY, Sato H, Kusam S, Sehra S, Toney LM, Dent AL (2005). Regulation of IL-10 gene expression in Th2 cells by Jun proteins. J Immunol.

[29] Ozawa T, James CD. Establishing intracranial brain tumor xenografts with subsequent analysis of tumor growth and response to therapy using bioluminescence imaging. J Vis Exp. 2010;(41):e1986. 10.3791/1986. 10.3791/1986 PMC3149989 20644517

[30] Zhou Y, Zhou Y, Shingu T, Feng L, Chen Z, Ogasawara M, Keating MJ, Kondo S, Huang P (2011). Metabolic alterations in highly tumorigenic glioblastoma cells: preference for hypoxia and high dependency on glycolysis. J Biol Chem.

[31] Compton LM, Ikonomov OC, Sbrissa D, Garg P, Shisheva A (2016). Active vacuolar H+ ATPase and functional cycle of Rab5 are required for the vacuolation defect triggered by PtdIns(3,5)P2 loss under PIKfyve or Vps34 deficiency. Am J Physiol Cell Physiol.

[32] Spitz DR, Sim JE, Ridnour LA, Galoforo SS, Lee YJ (2000). Glucose deprivation-induced oxidative stress in human tumor cells. A fundamental defect in metabolism?. Ann N Y Acad Sci.

[33] Song JJ, Lee YJ (2007). Differential activation of the JNK signal pathway by UV irradiation and glucose deprivation. Cell Signal.

[34] Zhu J, Zheng Y, Zhang H, Sun H (2016). Targeting cancer cell metabolism:the combination of metformin and 2-deoxyglucose regulates apoptosis in ovarian cancer cells via p38 MAPK/JNK signaling pathway. Am J Transl Res.

[35] Davis RJ (2000). Signal transduction by the JNK group of MAP kinases. Cell.

[36] Tournier C, Dong C, Turner TK, Jones SN, Flavell RA, Davis RJ (2001). MKK7 is an essential component of the JNK signal transduction pathway activated by proinflammatory cytokines. Genes Dev.

[37] Tsujimoto Y, Shimizu S, Eguchi Y, Kamiike W, Matsuda H (1997). Bcl-2 and Bcl-xL block apoptosis as well as necrosis: possible involvement of common mediators in apoptotic and necrotic signal transduction pathways. Leukemia.

[38] Nikoletopoulou V, Markaki M, Palikaras K, Tavernarakis N (2013). Crosstalk between apoptosis, necrosis and autophagy. Biochim Biophys Acta.

[39] Yamamoto K, Ichijo H, Korsmeyer SJ (1999). BCL-2 is phosphorylated and inactivated by an ASK1/Jun N-terminal protein kinase pathway normally activated at G(2)/M. Mol Cell Biol.

[40] Basu A, Haldar S (2003). Identification of a novel Bcl-xL phosphorylation site regulating the sensitivity of taxol- or 2-methoxyestradiol-induced apoptosis. FEBS Lett.

[41] Wei Y, Sinha S, Levine B (2008). Dual role of JNK1-mediated phosphorylation of Bcl-2 in autophagy and apoptosis regulation. Autophagy.

[42] Jefferies HB, Cooke FT, Jat P, Boucheron C, Koizumi T, Hayakawa M, Kaizawa H, Ohishi T, Workman P, Waterfield MD (2008). A selective PIKfyve inhibitor blocks PtdIns(3,5)P(2) production and disrupts endomembrane transport and retroviral budding. EMBO Rep.

[43] Recouvreux MV, Commisso C (2017). Macropinocytosis: a metabolic adaptation to nutrient stress in cancer. Front Endocrinol (Lausanne).

[44] Karin M, Liu Z, Zandi E (1997). AP-1 function and regulation. Curr Opin Cell Biol.

[45] Brown PH, Chen TK, Birrer MJ (1994). Mechanism of action of a dominant-negative mutant of c-Jun. Oncogene.

[46] Gopalan SM, Wilczynska KM, Konik BS, Bryan L, Kordula T (2006). Astrocyte-specific expression of the alpha1-antichymotrypsin and glial fibrillary acidic protein genes requires activator protein-1. J Biol Chem.

[47] Candolfi M, Curtin JF, Nichols WS, Muhammad AG, King GD, Pluhar GE, McNiel EA, Ohlfest JR, Freese AB, Moore PF (2007). Intracranial glioblastoma models in preclinical neuro-oncology: neuropathological characterization and tumor progression. J Neuro-Oncol.

[48] Shisheva A (2001). PIKfyve: the road to PtdIns 5-P and PtdIns 3,5-P(2). Cell Biol Int.

[49] Ikonomov OC, Sbrissa D, Shisheva A (2001). Mammalian cell morphology and endocytic membrane homeostasis require enzymatically active phosphoinositide 5-kinase PIKfyve. J Biol Chem.

[50] Choy CH, Saffi G, Gray MA, Wallace C, Dayam RM, Ou ZA, Lenk G, Puertollano R, Watkins SC, Botelho RJ (2018). Lysosome enlargement during inhibition of the lipid kinase PIKfyve proceeds through lysosome coalescence. J Cell Sci.

[51] Martin S, Harper CB, May LM, Coulson EJ, Meunier FA, Osborne SL (2013). Inhibition of PIKfyve by YM-201636 dysregulates autophagy and leads to apoptosis-independent neuronal cell death. PLoS One.

[52] Dukes JD, Whitley P, Chalmers AD (2012). The PIKfyve inhibitor YM201636 blocks the continuous recycling of the tight junction proteins claudin-1 and claudin-2 in MDCK cells. PLoS One.

[53] Ikonomov OC, Sbrissa D, Mlak K, Shisheva A (2002). Requirement for PIKfyve enzymatic activity in acute and long-term insulin cellular effects. Endocrinology.

[54] Shi L, Pan H, Liu Z, Xie J, Han W (2017). Roles of PFKFB3 in cancer. Signal Transduct Target Ther.

[55] Chesney J, Clark J, Lanceta L, Trent JO, Telang S (2015). Targeting the sugar metabolism of tumors with a first-in-class 6-phosphofructo-2-kinase (PFKFB4) inhibitor. Oncotarget.

[56] Clem B, Telang S, Clem A, Yalcin A, Meier J, Simmons A, Rasku MA, Arumugam S, Dean WL, Eaton J (2008). Small-molecule inhibition of 6-phosphofructo-2-kinase activity suppresses glycolytic flux and tumor growth. Mol Cancer Ther.

[57] Clem BF, O'Neal J, Tapolsky G, Clem AL, Imbert-Fernandez Y, Kerr DA, Klarer AC, Redman R, Miller DM, Trent JO (2013). Targeting 6-phosphofructo-2-kinase (PFKFB3) as a therapeutic strategy against cancer. Mol Cancer Ther.

[58] Dhanasekaran DN, Reddy EP (2017). JNK-signaling: a multiplexing hub in programmed cell death. Genes Cancer.

[59] Haldar S, Chintapalli J, Croce CM (1996). Taxol induces bcl-2 phosphorylation and death of prostate cancer cells. Cancer Res.

[60] Upreti M, Galitovskaya EN, Chu R, Tackett AJ, Terrano DT, Granell S, Chambers TC (2008). Identification of the major phosphorylation site in Bcl-xL induced by microtubule inhibitors and analysis of its functional significance. J Biol Chem.

[61] Sasi N, Hwang M, Jaboin J, Csiki I, Lu B (2009). Regulated cell death pathways:new twists in modulation of BCL2 family function. Mol Cancer Ther.

[62] Czabotar PE, Lessene G, Strasser A, Adams JM (2014). Control of apoptosis by the BCL-2 protein family: implications for physiology and therapy. Nat Rev Mol Cell Biol.

[63] Bassik MC, Scorrano L, Oakes SA, Pozzan T, Korsmeyer SJ (2004). Phosphorylation of BCL-2 regulates ER Ca2^+^ homeostasis and apoptosis. EMBO J.

[64] Sung KF, Odinokova IV, Mareninova OA, Rakonczay Z, Hegyi P, Pandol SJ, Gukovsky I, Gukovskaya AS (2009). Prosurvival Bcl-2 proteins stabilize pancreatic mitochondria and protect against necrosis in experimental pancreatitis. Exp Cell Res.

[65] Broker LE, Kruyt FA, Giaccone G (2005). Cell death independent of caspases: a review. Clin Cancer Res.

[66] Chambers JW, LoGrasso PV (2011). Mitochondrial c-Jun N-terminal kinase (JNK) signaling initiates physiological changes resulting in amplification of reactive oxygen species generation. J Biol Chem.

[67] Vanlangenakker N, Vanden Berghe T, Krysko DV, Festjens N, Vandenabeele P (2008). Molecular mechanisms and pathophysiology of necrotic cell death. Curr Mol Med.

[68] Song JJ, Lee YJ (2005). Cross-talk between JIP3 and JIP1 during glucose deprivation: SEK1-JNK2 and Akt1 act as mediators. J Biol Chem.

[69] Ahlstedt J, Fornvik K, Zolfaghari S, Kwak D, Hammarstrom LGJ, Ernfors P, Salford LG, Redebrandt HN (2018). Evaluating vacquinol-1 in rats carrying glioblastoma models RG2 and NS1. Oncotarget.

