# Supplementary material for: The JNK signaling pathway plays a key role in methuosis (non-apoptotic cell death) induced by MOMIPP in glioblastoma
Source: BMC Cancer. 2019 Jan 16;19:77. doi: 10.1186/s12885-019-5288-y (PMC6335761; doi:10.1186/s12885-019-5288-y)
Supplement: Supplementary file 1 — Figure S1. Increased phosphorylation of c-Jun, Bcl-2 and Bcl-xL are early events during MOMIPP-induced methuosis. (DOCX 117 kb) [file 12885_2019_5288_MOESM1_ESM.docx]

**Additional File 1**

**Fig. S1. Increased phosphorylation of c-Jun, Bcl-2 and Bcl-xL are early events during MOMIPP-induced methuosis.** A) U251 cells were treated for 4 h with MOMIPP or MOPIPP at a concentration of 10 μM and equal amounts of cellular protein were immunoblotted for phosphorylated and total c-Jun (A), p38 (B), or Bcl-2, Bcl-xL and BAD (C) as described in the Methods. All blots depicted are representative of similar results obtained in three separate experiments.
