# Supplementary material for: The JNK signaling pathway plays a key role in methuosis (non-apoptotic cell death) induced by MOMIPP in glioblastoma
Source: BMC Cancer. 2019 Jan 16;19:77. doi: 10.1186/s12885-019-5288-y (PMC6335761; doi:10.1186/s12885-019-5288-y)
Supplement: Supplementary file 2 — Figure S2.. Luciferase expression in stable U251-LUC cells. (DOCX 395 kb) [file 12885_2019_5288_MOESM2_ESM.docx]

**Additional File 2**

**Fig. S2. Luciferase expression in stable U251-LUC cells.** 1 x 10^5^ U251 or U251-LUC cells were seeded in 35 mm dishes with glass coverslips. After 24 h, the cells were washed with PBS and fixed with ice-cold methanol. Cells were incubated with 10% goat serum, followed by anti-luciferase antibody (1:100). Coverslips were washed and incubated with Alexa Fluor 568-labeled goat anti-mouse IgG (1:600, 1 h). Nuclear DNA was stained with DAPI for 5 min. Images were obtained with an Olympus IX70 inverted microscope. Scale bar: 50 μm.

**
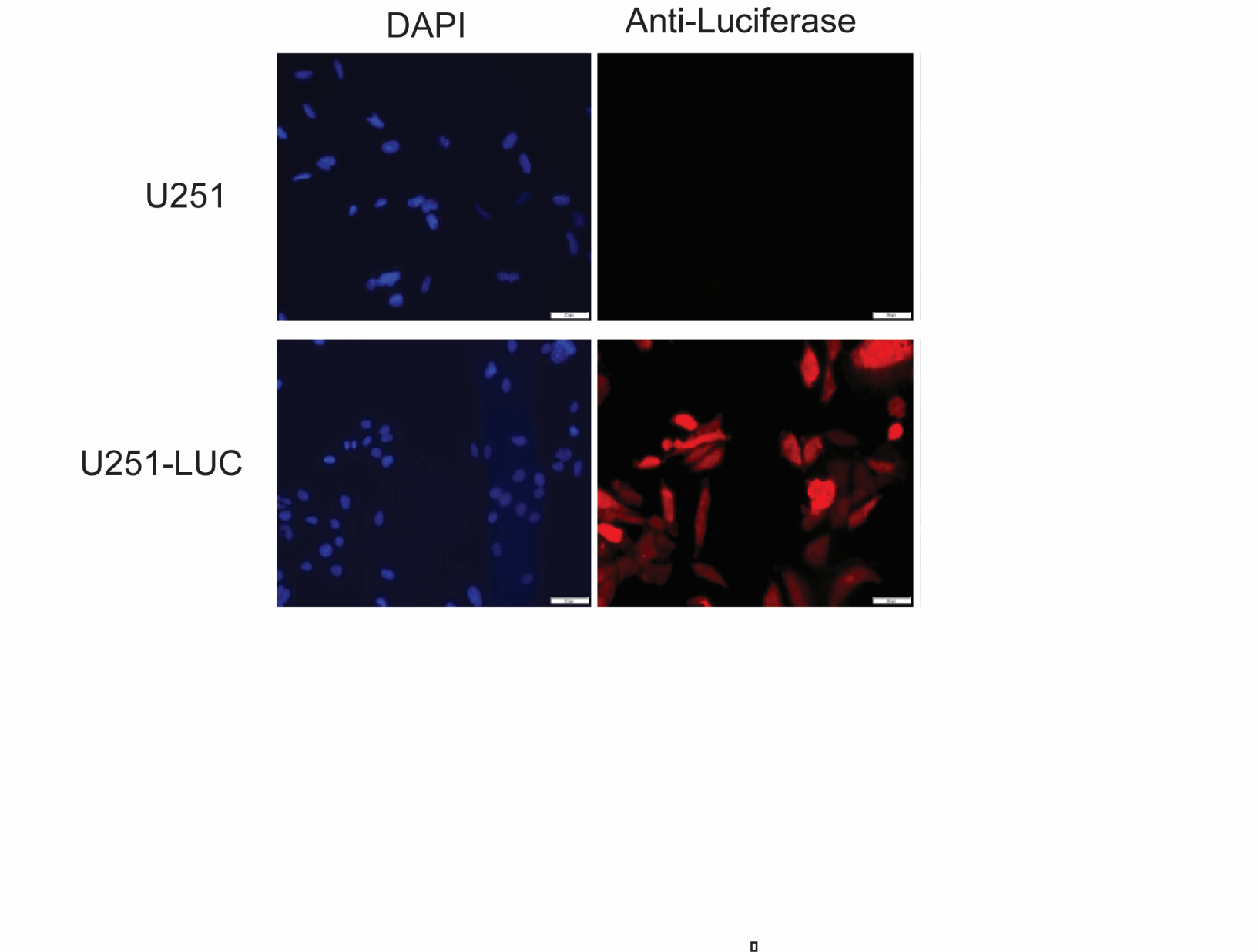
**
