# Supplementary material for: The JNK signaling pathway plays a key role in methuosis (non-apoptotic cell death) induced by MOMIPP in glioblastoma
Source: BMC Cancer. 2019 Jan 16;19:77. doi: 10.1186/s12885-019-5288-y (PMC6335761; doi:10.1186/s12885-019-5288-y)

**Additional File 3**

**Fig. S3.** **Long-term treatment with MOMIPP does not cause weight loss in nude mice**. The weights of individual control and MOMIPP-treated mice were determined at intervals during the antitumor efficacy study shown in Fig. 9B.


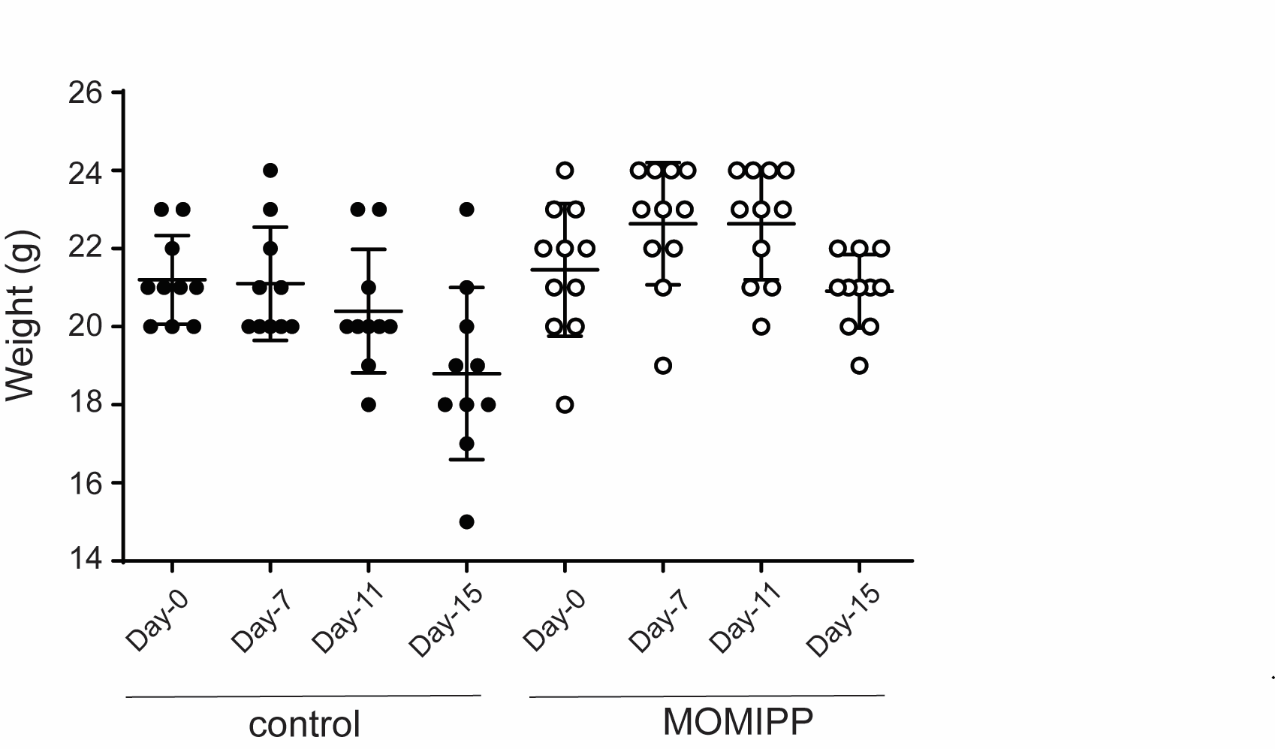

Supplement: Supplementary file 3 — Figure S3. Long-term treatment with MOMIPP does not cause weight loss in nude mice. (DOCX 159 kb) [file 12885_2019_5288_MOESM3_ESM.docx]
