# Supplementary material for: The JNK signaling pathway plays a key role in methuosis (non-apoptotic cell death) induced by MOMIPP in glioblastoma
Source: BMC Cancer. 2019 Jan 16;19:77. doi: 10.1186/s12885-019-5288-y (PMC6335761; doi:10.1186/s12885-019-5288-y)
Supplement: Supplementary file 4 — Table S1. Blood chemistry profiles obtained after treatment of mice for 15 d with MOMIPP or vehicle. (DOCX 13 kb) [file 12885_2019_5288_MOESM4_ESM.docx]

**Additional File 4**

**Table S1. Blood chemistry profiles obtained after 15 days of treatment with MOMIPP or vehicle in the study described in Fig. 9B**

| *Blood chemistry parameter* | *Unit* | *Control group (n=10)* | *MOMIPP group (n=11)* |
| --- | --- | --- | --- |
| Albumin (ALB) | g/dl | 3.96 ± 0.25 | 3.80 ± 0.57 |
| Alkaline phosphatase (ALP) | U/l | 79.7 ± 24.3 | 52.0 ± 17.0 |
| Alanine transaminase (ALT) | U/l | 108.4 ± 77.6 | 107.2 ± 78.4 |
| Amylase (AMY) | U/l | 871.0 ± 299.2 | 872.2 ± 143.2 |
| Total Bilirubin (TBIL) | mg/dl | 0.36 ±0.05 | 0.41 ± 0.03 |
| Blood urea Nitrogen (BUN) | mg/dl | 17.1 ± 5.2 | 20.2 ± 3.6 |
| Calcium (CA) | mg/dl | 11.23 ± 0.40 | 11.06 ± 0.48 |
| Phosphorus (PHOS) | mg/dl | 12.85 ± 1.28 | 11.45 ± 1.10 |
| Glucose | mg/dl | 197.0 ± 38.3 | 186.3 ± 23.0 |
| Na+ | mmol/l | 156.0 ± 4.1 | 156.4 ± 2.0 |
| Total Protein (TP) | g/dl | 5.59 ± 0.28 | 5.60 ± 0.67 |
| Globulin (GLOB) | g/dl | 1.61 ± 0.14 | 1.85 ± 0.30 |

Values are mean ± SD
