# Supplementary material for: The JNK signaling pathway plays a key role in methuosis (non-apoptotic cell death) induced by MOMIPP in glioblastoma
Source: BMC Cancer. 2019 Jan 16;19:77. doi: 10.1186/s12885-019-5288-y (PMC6335761; doi:10.1186/s12885-019-5288-y)
Supplement: Supplementary file 5 — Figure S4. MOMIPP-induced cell death is not prevented by a necroptosis inhibitor. (DOCX 471 kb) [file 12885_2019_5288_MOESM5_ESM.docx]

**Additional File 5**

**Figure S4.**  MOMIPP-induced methuosis is not prevented by a necroptosis inhibitor; necrostatin-1 (Nec-1). U251 cells were pre-treated with 100 µM of Nec-1 for 1 h, followed by incubation with 10 µM MOMIPP + Nec-1 (48 h) or 10 µM Shikonin + Nec-1 (6 h). Shikonin was used as a positive control, since it is known as a potent inducer of necroptosis [1]. Cell viability was assessed using the CellTiter Glo® ATP assay. Values are means ± S.D. from four replicates. Phase contrast pictures were taken after the indicated times. **A**. Nec-1 protected the cells from Shikonin-induced necroptosis. **B.** Necc-1 had no protective effect in the MOMIPP-treated cells, which still formed vacuoles and lost viability after 48 h. Scale bar: 20 μm.


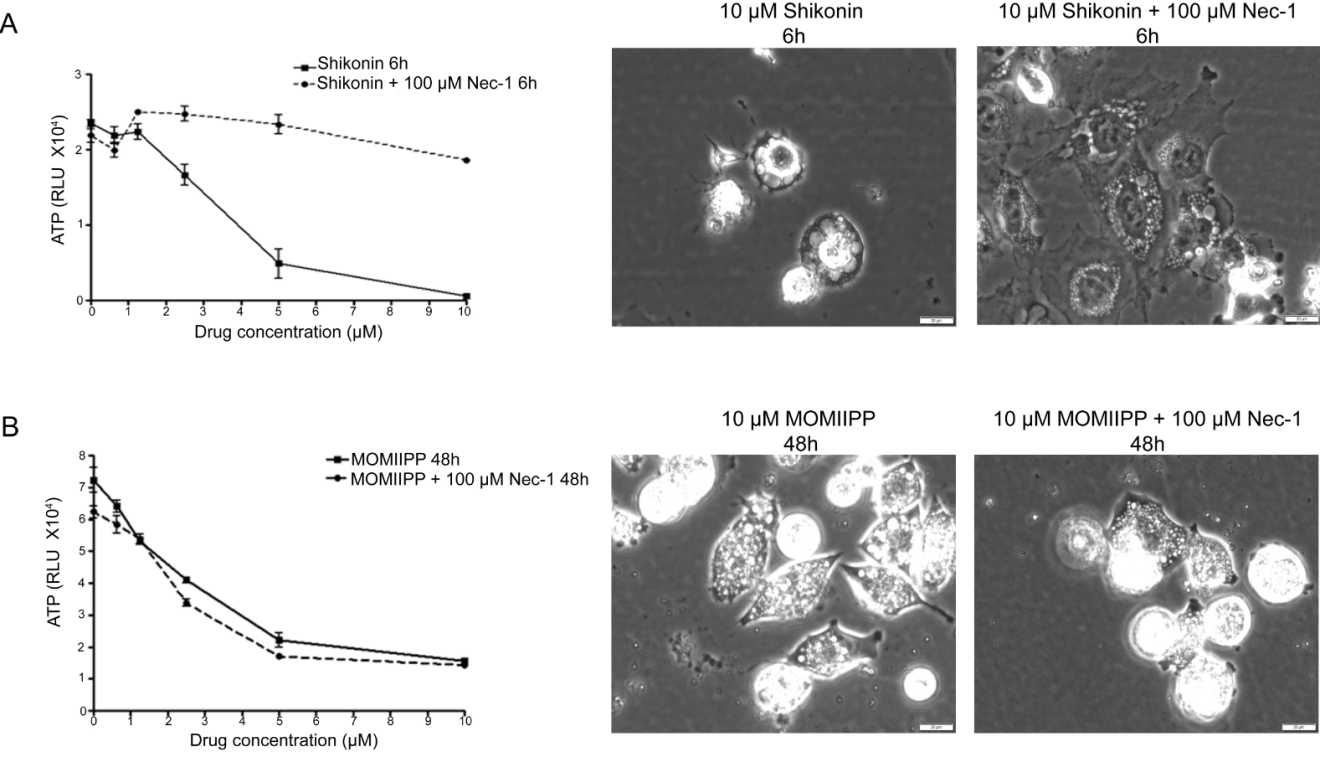


Reference:

1. Huang, C, Luo, Y, Zhao, J, Yang, F, Zhao, H, Fan, W, Ge, P. Shikonin kills glioma cells through necroptosis mediated by RIP-1. PLoS One 2013; 8(6): e66326.
